# Mapping the race between crop phenology and climate risks for wheat in France under climate change

**DOI:** 10.1038/s41598-024-58826-w

**Published:** 2024-04-08

**Authors:** Renan Le Roux, Carina Furusho-Percot, Jean-Charles Deswarte, Marie-Odile Bancal, Karine Chenu, Nathalie de Noblet-Ducoudré, Iñaki García de Cortázar-Atauri, Alexis Durand, Burak Bulut, Olivier Maury, Jérémie Décome, Marie Launay

**Affiliations:** 1 INRAE, AgroClim, 84914 Avignon, France; 2 ARVALIS - Institut du Végétal, Villiers-le-Bâcle, France; 3 Université Paris-Saclay, INRAE, AgroParisTech, UMR Ecosys, 91120 Palaiseau, France; 4 Queensland Alliance for Agriculture and Food Innovation, The University of Queensland, 13 Holberton Street, Toowoomba, QLD 4350 Australia; 5 Commissariat à l’Energie atomique et aux énergies alternatives (CEA), Université Paris-Saclay, LSCE/IPSL, 91191 Gif-Sur-Yvette, France

**Keywords:** Climate-change impacts, Abiotic

## Abstract

Climate change threatens food security by affecting the productivity of major cereal crops. To date, agroclimatic risk projections through indicators have focused on expected hazards exposure during the crop’s current vulnerable seasons, without considering the non-stationarity of their phenology under evolving climatic conditions. We propose a new method for spatially classifying agroclimatic risks for wheat, combining high-resolution climatic data with a wheat’s phenological model. The method is implemented for French wheat involving three GCM-RCM model pairs and two emission scenarios. We found that the precocity of phenological stages allows wheat to avoid periods of water deficit in the near future. Nevertheless, in the coming decades the emergence of heat stress and increasing water deficit will deteriorate wheat cultivation over the French territory. Projections show the appearance of combined risks of heat and water deficit up to 4 years per decade under the RCP 8.5 scenario. The proposed method provides a deep level of information that enables regional adaptation strategies: the nature of the risk, its temporal and spatial occurrence, and its potential combination with other risks. It’s a first step towards identifying potential sites for breeding crop varieties to increase the resilience of agricultural systems.

## Introduction

Food security is the second sustainable development goal defined by the United Nations. This objective is increasingly harder to achieve due to the fast population growth and because climate change is constraining the production of cereal crops, which provide two-thirds of human caloric intake^1^.

Along with maize and rice, wheat (*Triticum* spp.) is one of the most important cereal crops, totalling around 220 Mha surface and 750 Mt grain production (average 2015–2020, FAO Stat). It provides about 20% of the calories and 20% of the protein for daily human consumption. In this context, France is one of the main wheat producing (5th) and exporting (4th) countries, wheat being grown on about 5 Mha with high average yields achieving 7t.ha^−1^, whereas European and global wheat yields average 5.5 t.ha^−1^ and 3.5 t.ha^−1^ respectively (average 2015–2020, FAO Stat).

Global temperature increase, precipitation patterns change, as well as greater frequency of extreme events such as floods, droughts and heatwaves, already strongly affect wheat cropping across the word^1–3^. In Europe, there is a trend towards a slowdown in yield increases from the 1990s onwards, an increase in absolute yield variability^4^, and a slight increase in loss propensities for most annual crops^5^. The critical environmental factors contributing to the stagnation of wheat yields at the French national level include heat stress during grain filling and drought during the stem elongation and grain-filling stages, as identified by^6^. Additionally, water excess and crop cycle shortening also contribute to yield stagnation, with a high distribution heterogeneity at the subnational level^7^. Simultaneous occurrence of multiple unprecedented events also led to significant yield losses, as observed in 2016 due to both anomalous warm temperatures during late autumn and abnormally wet conditions and reduced solar radiations in the following spring^8^.

Recent modeling studies at the global scale projected substantial losses at lower latitudes and gains at high northern latitudes especially for wheat, favoring peak productivity zones shift poleward^9,10^. Underlying drivers include shorter growing seasons due to warming, and physiological disorders associated with a significant increase in extreme heat and drought events. Those unfavorable effects may be efficiently counterbalanced by the "fertilizing" effect of atmospheric CO_2_ concentration on biomass production^11^ and adaptation levers such as sowing date and cultivar choices^9,12^. However, yield gains may also be tempered by major changes in pathogen assemblages and increased disease severity^13^. Modeling approaches^14^ mainly describe climate trends effects (e.g. average increase over a growing season or year in temperature) smoothing the variability of weather variables and extreme events (heat waves, excess water, droughts, etc.). These extreme events are nevertheless projected to become more frequent and intense, and their omission in models can lead to underestimation of yield losses^15^. France is located on the boundary between the Mediterranean and the oceanic climatic zones, which are projected to shift and extend northwards^16^ thus impacting current production areas. This geographic position places the country in a strong spatio-temporal climatic gradient which must be taken into account in the design of adaptation strategies for wheat cropping systems.

Long-term adaptations include breeding of crop varieties^17^ that escape, tolerate or resist to more frequent and intense seasonal abiotic and biotic stresses^18^. Breeding is long-lasting process that implies anticipation of future climatic conditions, in order to select the more relevant phenotypic traits to face climate change. Moreover, in countries like France and Australia, which may exhibit contrasted regional phenoloclimates^19,20^, new crop varieties must be regionally suited to minimize abiotic stresses hindering crop productivity^12,21^. Indeed, heat, cold or even frost, drought or water logging may have damageable effects on crop functioning during sensitive phenological phases of the crop cycle. The impact of climate change on agricultural productivity is a significant concern worldwide, and numerous studies have investigated the effects of climate variability on crop yields^6,22^. However, much of this research has focused on broad-scale analyses that provide limited spatial resolution and fail to account for the plants’ phenology that is also impacted by specific agro-climatic conditions of individual regions.

In this context, it is necessary to develop methods allowing better (1) identification of climate hazards especially at critical phenological phases, (2) characterisation of phenological shifts in crop sensitive phases under global warming, (3) assessment of spatio-temporal climatic trends including for extreme events, (4) quantification of potential damages caused to crops, (5) identification of ecoclimatic clusters sharing similar regional risks.

In the present study, we propose a methodology considering the spatio-temporal evolution of climatic conditions for a crop (wheat) and apply it to France, a leading wheat-producing and wheat-exporting region in the world. We focus on the conditions likely to prevent the proper and complete development of the crop cycle. In a first step, wheat ecoclimatic indicators (i.e. agro-climatic indicators calculated for a given phenological phase) are built to provide information about the effect of climate events or conditions on plant processes during specific phenological periods^23^. Moreover, indicators are normalized to fit a unique scale of comparable damages irrespective of their nature (e.g. cold, heat or water stress). Those indicators are all defined to express wheat vulnerability to climate change and combine climate hazards, crop exposure and sensitivity. In a second step, we consider the regional climatic variability in France^19^. Thus, we propose a simple unsupervised classification method to divide the territory into sub-regions using the wheat ecoclimatic indicators. Last, we highlight spatial patterns of temporal evolution for wheat growing conditions between now and the end of the century, and we analyse and discuss the most threatening future compound events, specifically the interplay of heat and water stress. Additionally, we propose strategies aimed at the identification and selection of phenotypes that exhibit local adaptability to the anticipated future climatic conditions.

## Methods

### Climate data

**Figure 1:**
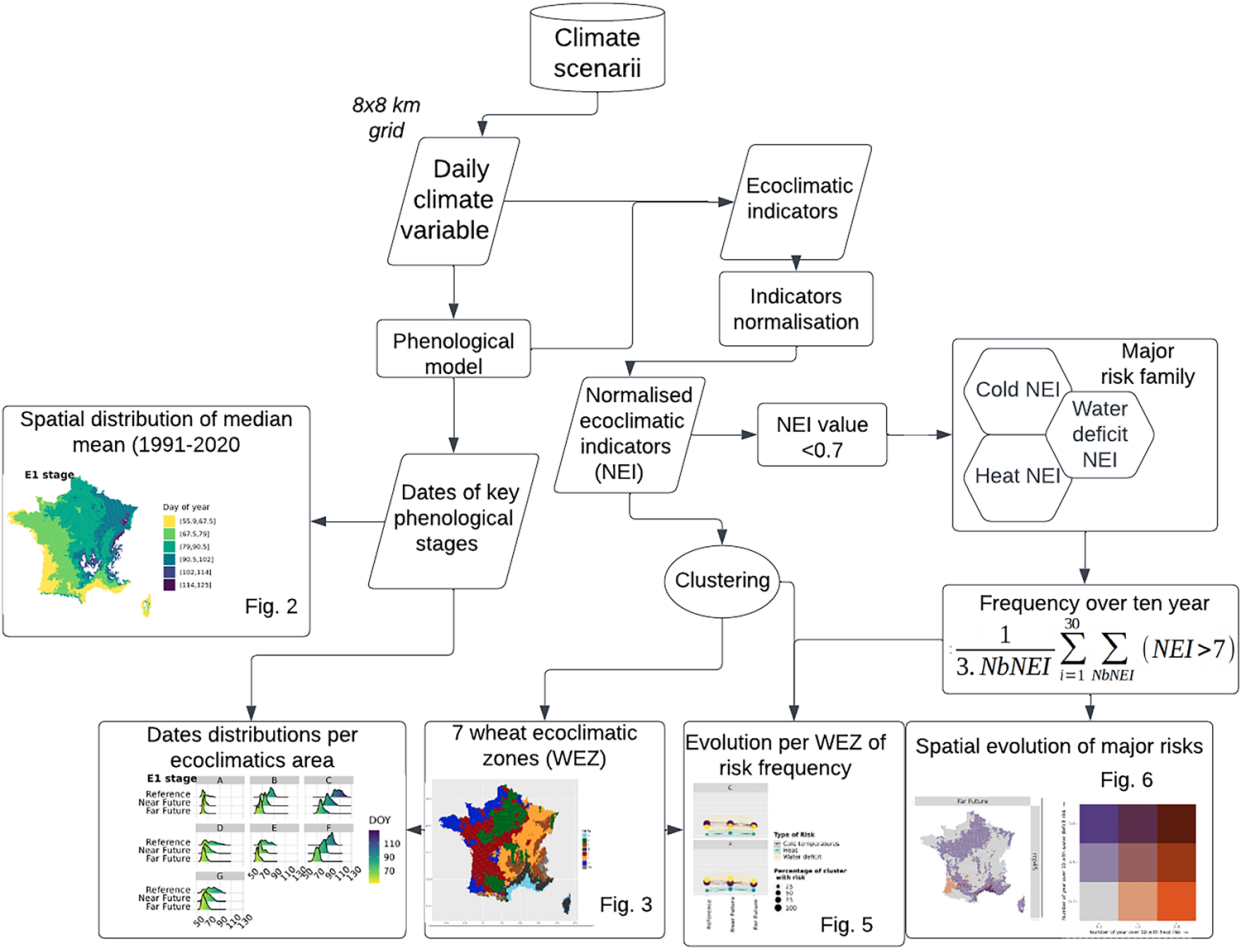
Workflow of the analysis using classic flowcharts as symbols for each dataset, process and results. NEI: normalized ecoclimatic indicator, NbNei: the number of indicators per major risk family, WEZ: wheat ecoclimatic zone, 3.Nbei: 3 climate models x NbNei.

For the construction of the indicators and the phenological model, we use two climate databases. A first 2012–2021 database corresponding to the measurement period for phenology observations in the Epiphyt database is composed of SAFRAN reanalysis. SAFRAN is a method allowing the production of historical climatic data at high spatial resolution (8 km) specific to France. All the details of the processing chain can be found in Ref.^24^. The SAFRAN database is handled to run and evaluate the phenological model upstream (Fig. 1).

A second climatic database is used for historical and future simulations, with data from climate model outputs that are extracted from Drias portal^25^, which is the most recent data from regionalization of climate models over France. The Adamont bias correction method^26^. produces results on the same spatial grid as SAFRAN (8 km), and has been evaluated in comparison with the SAFRAN reanalysis itself, showing similar or even better evaluation metrics than alternative methods^26^. Three Global Circulation Models—Regional Climate Model (GCM-RCM) pairs are utilized, (CNRM-Aladin63, CNRM-Racmo, and EC-Earth Racmo). The choice to keep only these three models is pragmatic, as they are the only ones including evolving aerosols and producing a global radiation variable, necessary for the calculation of some indicators. The periods selected are the reference period (1991–2020), near (2041–2070) and far (2071–2100) futures, for two emission scenarios RCP (Representative Concentration Pathways) 4.5 and 8.5.

In order to observe the spatial evolution of wheat growing opportunities in France as a result of climate change, SAFRAN and DRIAS data were downloaded and used to calculate the indicators for the entire French territory, including areas that do not support wheat growth (e.g. at high altitudes), but for evaluation of the reliability of phenology simulations over the historical period, areas where the phenological cycle has not been reached are excluded.

### Wheat variety and phenological dataset and model

We chose the bread wheat variety Talent, representing a common phenological pattern in France, with medium precocity, large cold requirements and grown in all wheat producing regions amongst the country. The sowing date was set to 15th October which corresponds to the average date of winter wheat sowing in France (French FranceAgriMer Observatory Céré’Obs https://cereobs.franceagrimer.fr). The Epiphyt database, consisting of thousands of observed wheat stages over the country since 2014, with no information on sowing date nor cultivar, is handled to evaluate the accuracy of the phenological model. This database is provided through the TEMPO data portal (https://data.pheno.fr/). The phenological model relies on a photo-vernal thermal time approach. It assigns fixed developmental time intervals between stages defined as emergence (EM) corresponding to BBCH 10, ear 1 cm (E1) corresponding to BBCH 30, flag leaf (FL, BBCH 39), anthesis (AN, BBCH 65) and grain maturity (GM, BBCH 89). Development time is calculated by accumulating the daily thermal time increment according to the growing degree days approach (GDD in °C days), with a base temperature of 0 °C^27^, slowed by sub-optimal photoperiod conditions, and/or by non-compliance with vernalisation requirements^28^. Phenology parameters’ set include a fixed photoperiod sensitivity but cultivar specific vernalization and thermal requirements that have been set for the Talent variety (see Supplementary Fig. S2).

### Ecoclimatic indicators for wheat

The method for building ecoclimatic indicators has three main steps ^23^: (1) dividing the crop cycle into phenological phases corresponding to the different sensitive periods, (2) defining raw ecoclimatic indicators for these phenological phases, and (3) normalizing these ecoclimatic indicators through ecophysiological or agronomic response functions in order to allow meaningful comparison of potential damages related to the different indicators. A normalized index equal to 1 means no stress effect during the crop phenological phase, while a value of 0 represents a maximum value of stress. Two functions are used for the normalization: a sigmoid and a negative exponential ones^23^ (see Supplementary Fig. S1). All those steps rely on the scientific literature and expert assessment. The selected wheat phenological phases are sowing (SO) to emergence (EM), EM to ear 1 cm (E1), E1 to flag leaf (FL), FL to anthesis (AN) and AN to grain maturity (GM).

The elementary indicators (before normalization) characterize water deficit, water excess, heat or cold temperatures, that may impede wheat functioning over a critical threshold. Thresholds are based on literature references and are defined according to the phenological phases of wheat during which the relative climate hazards occur. The ecoclimatic indicators and their normalization are detailed below and in Supplementary Table S1, and their calculation was carried out with the SEASON system^29^.

At the beginning of the crop cycle, both seed moistening and coleoptile growth are closely linked to soil moisture. A 5-day flood is enough to kill the seed^30^, whereas germination is delayed if the seedbed dries out, without impairing the seed viability as long as the seed has not already imbibed water. If however the soil water content has been high enough to allow seed moistening, the time for germination after moistening reduces the seed viability, which reduces the germination rate^31,32^. Moreover, during the initial seedling stage, low daily average temperatures below 2 °C (during more than 2 successive days) delay emergence and induce poor vigor plantlets^33^. Daily minimum temperatures below − 5 °C destroy coleoptiles, and this lethal effect is increased by the duration of the freezing event^34^. After emergence and till the end of tillering, low average temperatures below 5 °C reduce leaf initiation, growth rate and biomass^35^, while a prolonged period with daily average temperatures below 0 °C reduce tillers number, provoke an uneven stand establishment and kills stem apex^36^. Frost events relying on minimum temperatures below -8 °C kill the plantlet^34^. On another hand, the vernalization process, which is essential for flowering initiation, is paused when the average temperature exceeds a threshold between 16 and 18 °C, with strong interaction with the photoperiod, as short days can replace cold temperature response^27,37^. The daily minimum temperature for vernalization to occur is assessed at 0 °C^38^, while the duration necessary to attain vernalization saturation (i.e. the duration for which the final leaf number is the lowest^39^) varies among cultivars^37^. Heat events may thus stop or delay the vernalization process, or even prevent flowering to occur. Vernalization may be reversed, leading to ‘devernalization’, as experimentally shown by Dubert et al.^40^, for controlled conditions with constant temperatures above 20 °C.

During the tillering phase, warm nights accelerate and increase tiller mortality, leading to a lower spike establishment^41^. Plants exposed to warmer nights have a greater respiratory activity, which results in carbon loss and less assimilates available for plant growth^42^. Higher tiller mortality was observed for minimum temperatures above 12 °C between the beginning of stem elongation and 10 days after anthesis^34,41,43^.

During elongation and booting, cold daily minimum temperatures below 0–4 °C ^21,34^ curb stem elongation and frost (temperatures below − 5 to − 8 °C) limits the internode extension, denatures the spikelets, reduces assimilate transport, restricts the dry matter accumulation, and causes a significant reduction in grain yield^36^. The pollen mother cells division is particularly sensitive to successive cold days with low radiation (< 200W/m^2^), which can significantly reduce pollen fertility. Minimum temperatures below 4 °C 10 days around meiosis may thus reduce grain number^21^. This sterility is increased for temperatures below 0 °C^30^. Till flowering, very cold average temperatures between 0 and − 2 °C delay floret growth and induce leaf chlorosis and wilting, and also denature spikelet^44,45^.

Reproductive and grain filling phases are also extremely sensitive to heat and even a mild heat event during these stages can significantly reduce grain number and/or grain weight^46^. High temperatures during pollen mother cells division, can substantially sterilize the developing wheat pollen, and thus affect the grain set^47^. Heat events responsible for this fertility drop correspond to daily maximum temperatures above 30 °C^34,47–49^. Post-anthesis heat reduces grain yield by (i) shortening the grain filling duration^50,51^ and (ii) limiting assimilate synthesis and translocation to the grain sink ^52^. A strong correlation was observed and a linear regression established between the number of post-flowering days with maximum temperature > 30 °C and individual grain weight^46^. Simultaneously, very cold minimum temperatures between − 2 and − 6 °C reduce the number of grains and spikes, involve incomplete fruit setting and decrease grain weight^53^. Nevertheless, those thermal conditions remain very scarce.

Although drought may affect wheat growth during all phenological stages^54^, reproductive and grain-filling phases are extremely susceptible to drought^55^, which may cause substantial reduction in grain yield^56^. Even a brief episode of drought during meiosis of pollen mother cells may result in pollen sterility, thus reducing the grain set^54^. Water scarcity during early grain development curtails potential grain size by reducing the rate and duration of grain filling^57^, causing substantial decrease in grain dry weight^58–60^. Prolonged mild drought at flowering and grain filling can drastically reduce grain yield by 58–92% in studies reviewed by^54^.

To illustrate the construction of these elementary indicators and their normalization, let us take the example of cold temperature on seedlings. As research findings revealed that low temperatures around 5 °C, imposed at the seedling stage during 12 days, resulted in a 45% decrease in the photosynthetic rate as compared with control plants at 20 °C^35^, a “number of cold days with daily average temperature below 5 °C” indicator was defined during the phase emergence (EM) to ear 1 cm (E1). In that case, the normalization function is exponential leading to a normalized value of 0.55 since 15 cumulated cold days (Table S1).

For drought, while sensitive periods can be established from the literature, setting critical stress thresholds is not trivial, in particular because drought effects are highly dependent on soil characteristics. We have therefore chosen to use a purely climate-dependent indicator. First we compute the cumulative difference between daily rainfall and potential evapotranspiration (PET) between January 1 of the sowing year (year preceding the main growing season) and the end of the growing season for a proper initialization of the atmospheric water balance. Then we compute the sum of these daily value during each stages. For each drought indicator, the critical threshold for the normalization function is set at the value of the 1st quartile of its distribution (for all the spatial grid cells and over the reference period 1991–2020).

### Workflow analysis

Figure 1 presents the whole workflow, which is detailed below.

Phenological stages and ecoclimatic indicators calculation

First, for all spatial grid cells, the dates of the different phenological stages are simulated by the phenological model. Then the normalized ecoclimatic indicators are calculated per grid cell, per year and per phenological phase.

Clustering method

The sub-regionalization of the climatic risks related to wheat cultivation is carried out from the indicators calculated over a reference reference period (1991–2020) from the studied GCM-RCM climatic models using data from DRIAS. For this reference30-year period and for each normalized indicator, the number of occurrences of risk years has been calculated. A year with risk is a year in which the value of the normalized indicator is less than 0.7, whatever the indicator. For each pixel and each indicator, a score between 0 and 30 is obtained (30 means that the normalised indicator is less than 0.7 all years). If the value is 0 on all pixels in all years (risk never occurs anywhere), the indicator is removed from the clustering method. An unsupervised *K*-means classification^61^ is then performed on the remaining indicators, with the number of clusters set at 7 by the Elbow method^62^.

Future risks

For the 7 clusters identified, the same indicators are computed for the near future (2041–2070) and far future (2071–2100) for the different climate models. The model variability is characterized by the 95% and 5% quantiles.

Aggregation

To provide an overview of risk trends from the past to the future, all results are aggregated. First, all normalized indicators are calculated for each 8-square-kilometer cell, then summed over the entire period to give the number of occurrences over 30 years within each studied timeframe (1991–2020; 2041–2070 and 2071–2100) and RCP scenario. The indicators are then grouped by major risk categories (cold temperatures, water excess, water deficit, and heat).

For each major risk family (cold temperatures, heat and water deficit), we count the number of years with a risk and the number of indicators present in this risk family, then the annual risks for each family are divided by the number of indicators to have equivalent weights.

For each risk category, an average frequency of stress occurrence is calculated as the ratio between the number of occurrences of stress (indicator < 0.7) per season and the number of indicators in the considered risk category (see Supplementary Fig. S2).

## Results

### Spatial distribution of the occurrence of key phenological phases in France in the past

The phenological model with standards genotype and sown date agrees with observations from the Epiphyt database. The model simulates ear 1 cm (E1), flag leaf (FL) and anthesis (AN) stages with an average error of 6.5, 3.9 and 5.0 days, and a RMSE of 13.1, 8.1 and 7.6 days respectively, comparable to observations’ errors (see Supplementary Fig. S3). This result is also consistent with other calibrations in which the error was about 6–9 days for the E1 stage and 6 to 7 days for the FL stage ^63^.

**Figure 2:**
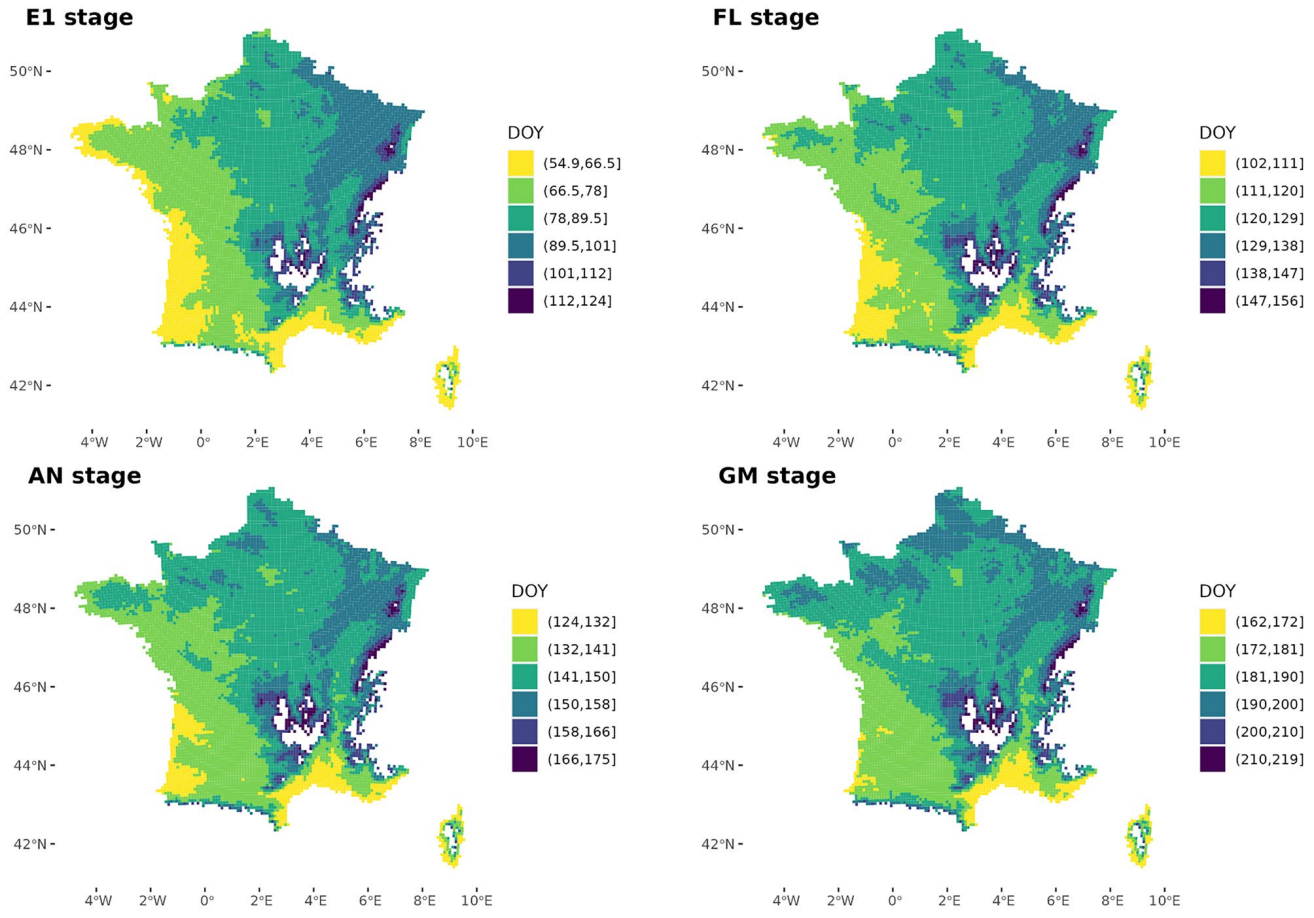
Spatial distribution of wheat key phenological stages across France, during the reference period 1991–2020: ear 1 cm (E1), flag leaf (FL), anthesis (AN) and grain maturity (GM). Colors correspond to classes of median dates expressed as day of the year (DOY) at which the simulated stage occurred [minimum median date for the earliest cell, maximum median date for the latest cell].

Winter wheat development is mainly driven by temperature. The climate heterogeneity in France generates spatial variability in the phenological stages of wheat (Fig. 2). Phenological stages may have 40–60 days of regional difference. For example, the ear 1 cm stage (E1 stage), can occur between early March (Western and Mediterranean coasts) and mid-April (North-east), while the grain filling phase currently occurs between early May and mid-June in the Mediterranean zone, and between early June and July in the traditional breadbasket (northern and eastern regions). There is a certain spatial stability between phenological stages (Fig. 2), with some variations as e.g. the initial precocity in the western regions disappears as the crop develops. This is due to a continental effect on phenology, as winters in France are milder near the coastlines. As the season progresses, the effect of latitude becomes more important. The Mediterranean area is the earliest throughout the cycle, while the mountainous and the northeast areas remain the latest.

The choice of a bread wheat variety with medium precocity for the simulations, allows to represent large inter and intra-regional differences, but may mask extremely early or late dates of sensitive stages. For this reason, in this study, we reason in terms of the frequency of years with a proven risk.

### Clustering French wheat zones according to phenology and climate risks

**Figure 3:**
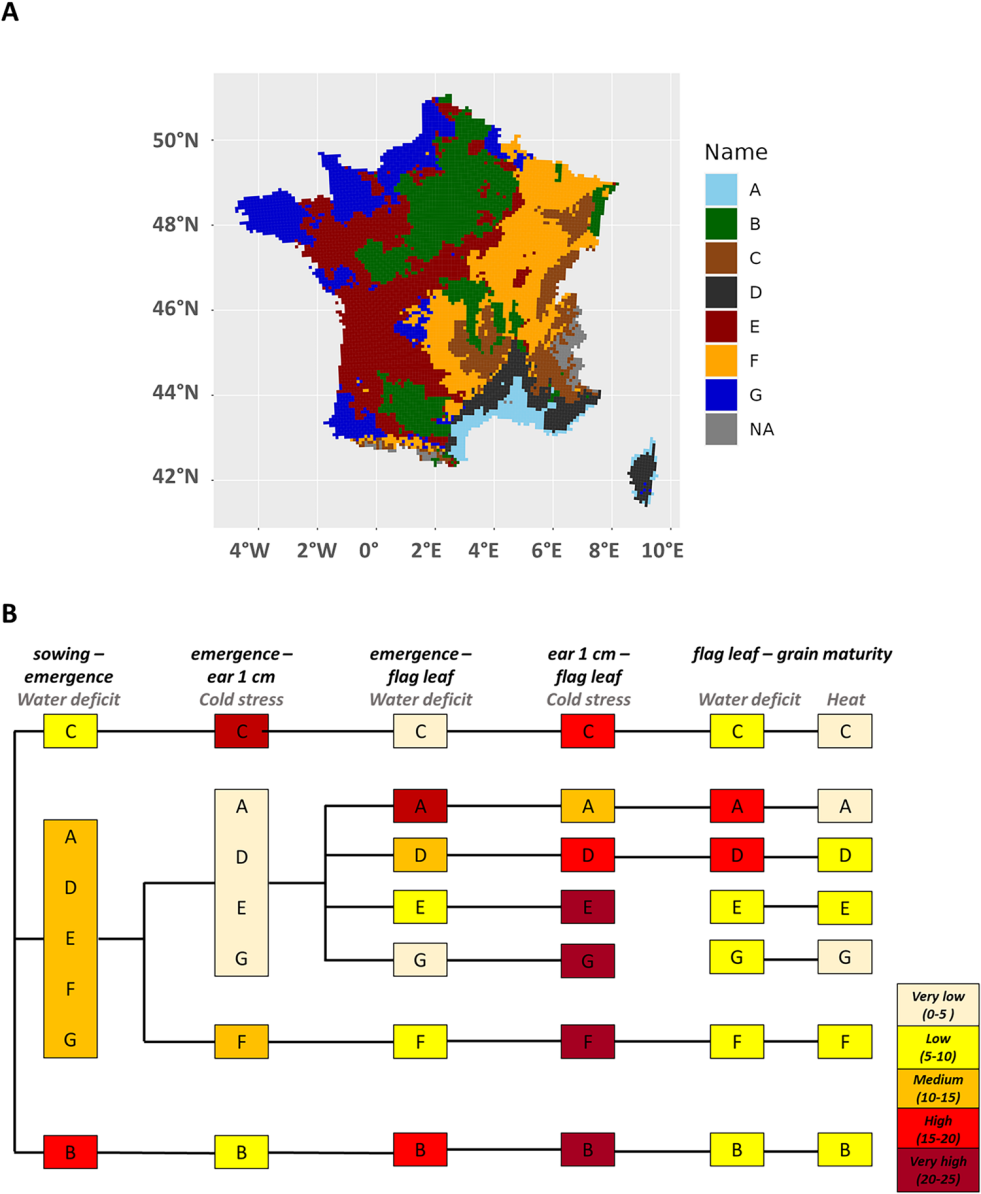
Spatial distribution (**A**) and main characteristics (**B**) of wheat ecoclimatic zones in France during the reference period (1991–2020). In (**B**), intensity of the main climate hazard for the different phenological phases is presented for each ecoclimatic zone (nude: very low intensity below 5 over 30 years; yellow: low intensity between 5 and 10 over 30 years; orange: medium intensity between 10 and 15 over 30 years; red: high intensity between 15 and 20 over 30 years; dark red: very high intensity between 20 and 25 over 30 years).

The spatial distribution of ecoclimatic indicators for wheat allows French wheat regions to be characterized according to climate risks faced by crops. The clustering method implemented on wheat ecoclimatic indicators provides a spatial description for the historical period 1991–2020 (Fig. 3).

The clusters identify 7 French wheat ecoclimatic zones (WEZ), named randomly from A to G.

- WEZ A along the Mediterranean and Corsica coast (Fig. 3a), is the least threatened by cold temperatures between emergence and flag leaf stage, but twice to three times more prone to water deficit from emergence to grain maturity, than in other WEZs (Fig. 3b). WEZ D, located in the Mediterranean hinterland and inland Corsica, exhibits a degraded Mediterranean climate, with less water deficit throughout the crop cycle, less heat stress from flag leaf to grain maturity, but even more cold stress during the juvenile stages.
- WEZ C is located in the high-altitude zone. This WEZ stands out from the other WEZs by a high frequency of freezing temperatures between emergence and ear 1 cm stage.
- WEZs E and G, located in the westernmost area (Fig. 3a), experience intermediate drought stress between sowing and emergence and very frequent cold temperatures between ear 1 cm and flag leaf stages. In the southern part of this area (WEZ E, except southwestern tip), heat stress occurs more frequently than in the northern part (WEZ G) from flag leaf to grain maturity. In WEZ B, subject to a less pronounced oceanic influence, water deficit may occur more often during juvenile stages (from sowing to flag leaf) than in WEZs E and G (Fig. 3b). Cold stress is also more common in WEZ B to ear 1 cm stage.
- WEZ F is further east within a continental climate, and exhibits more frost risks during the juvenile stage than other WEZs (10 to 15 over 30 years) except WEZ C, and less drought and heat risks over the whole crop cycle.

Overall, the cold and water stress indicators are the most discriminating between WEZs during the juvenile phases (between emergence and flag leaf), while heat and water stress take over between flag leaf and grain maturity stages. Water deficit is expected to alter more growth and yield elaboration after the flag leaf stage. Frost is particularly threatening at the beginning of the growing cycle in the mountain WEZ C, while all WEZs are affected by the risk of cold temperatures from the ear 1 cm to the flag leaf stage. Finally, between the flag leaf and grain maturity stages, heat has a fairly uniform and low impact among the WEZs. The south-western part of the country, under oceanic influence (WEZ E), experiences more frequent heat stresses because higher temperatures of lower latitudes combine to the relative later anthesis and grain maturity stages (cwater deompared to the Mediterranean WEZ A).

For those recent climatic conditions, warmer winters do not threaten vernalisation during the juvenile phase, nowhere in France. Cold or very cold temperatures do not distinguishWEZs after the flowering stage, and neither do the excess of water indicators throughout the crop cycle.

### Future evolution of the spatial and temporal distribution of wheat phenology

**Figure 4:**
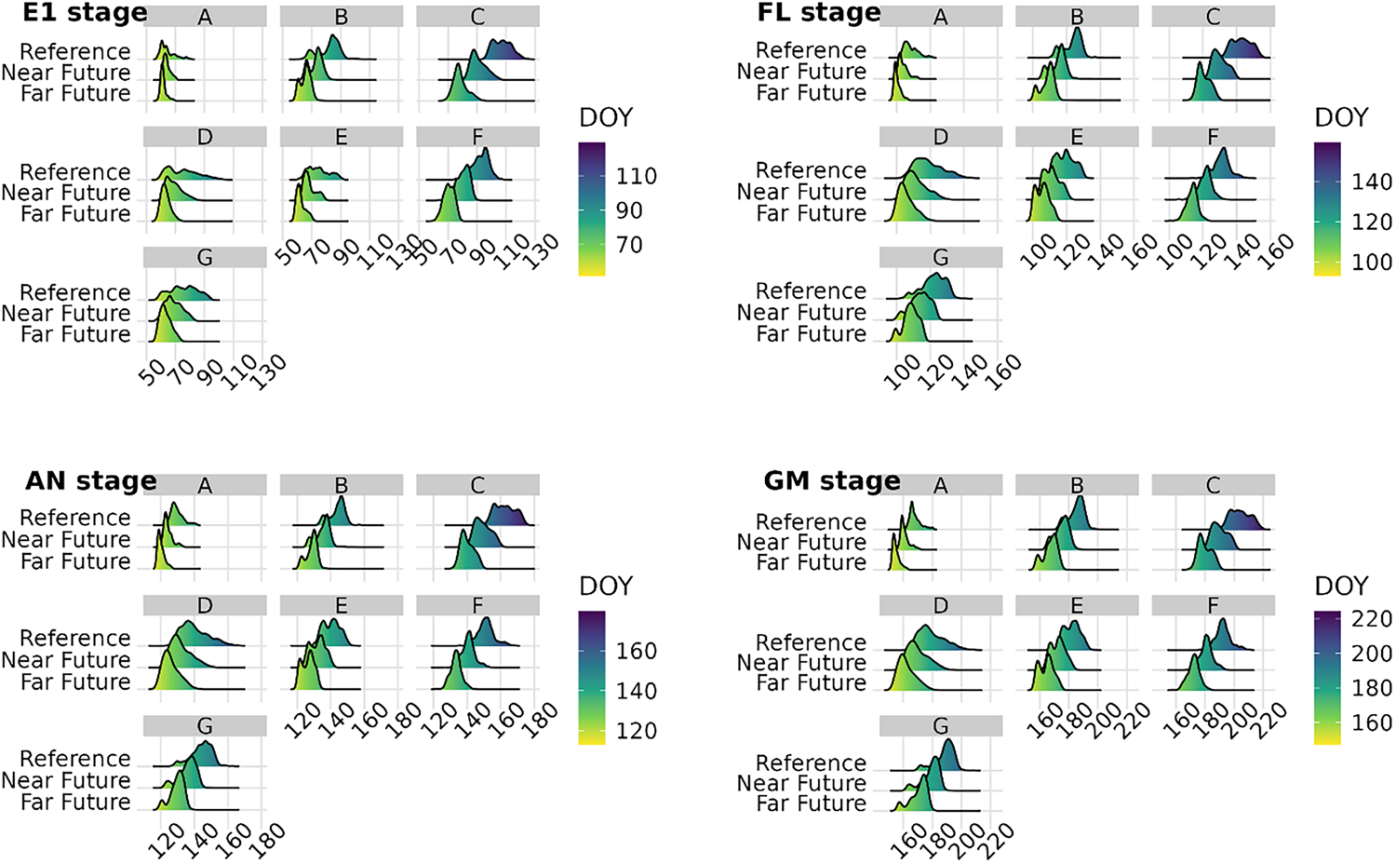
Distribution of wheat main phenological phases in the 7 French wheat ecoclimatic zones (WEZs A to G) for the reference (1991–2020), near (2041–2070) and far (2071–2100) future periods under RCP 8.5 scenario. EM-E1: emergence to ear 1 cm; E1-FL: ear 1 cm to flag leaf; FL-AN: flag leaf to anthesis; AN-GM: anthesis to grain maturity; DOY: day of the year.

Since phenology mainly depends on temperature, sensitive phenological phases are likely to shift with climate change. Figure 4 presents the projected evolution of the wheat phenological phases for the different WEZs in the near and far futures with scenario RCP 8.5 (see Supplementary Fig. S4 for RCP4.5).

An acceleration of the wheat phenology is projected, with earlier key phases in every WEZs. However, the situation varies considerably across WEZ: the greatest acceleration is projected in the most continental and mountainous WEZs (WEZ C and F), with shifts of about 2 weeks under RCP 4.5 scenario and 3–4 weeks under RCP 8.5 in WEZ C, while a shift of about 10 days under RCP 4.5 and 2 to 3 weeks under RCP 8.5 is expected in WEZ F in the far future. The ear at 1 cm stage is the stage most advanced by warming in these regions, with vernalisation facilitated by the reduction of excessively low temperatures. In the Mediterranean WEZ A, the phases shift less, from 0 to 3 days (in the near and far future respectively) for the youngest phase under RCP 4.5 scenario, to 2 weeks for the grain-filling phase in the far future under RCP 8.5. Grain maturity stage is the most advanced stage for all WEZs except mountainous and continental WEZs C and F, its advance ranging from 1 to 2 weeks in the near future under the RCP 8.5 scenario (ranging from 5 to 10 days under RCP 4.5, depending on the WEZ), and from 2 to more than 3 weeks in the far future for the same scenario. Overall and for both scenarios, the phases remain the earliest in the southernmost WEZs A and D.

### Future evolution of the climate risks in the 7 French wheat ecoclimatic zones

While climate change exacerbates heat stress, water deficit is partially avoided by the accelerating phenology of the crop in response to warming.

**Figure 5:**
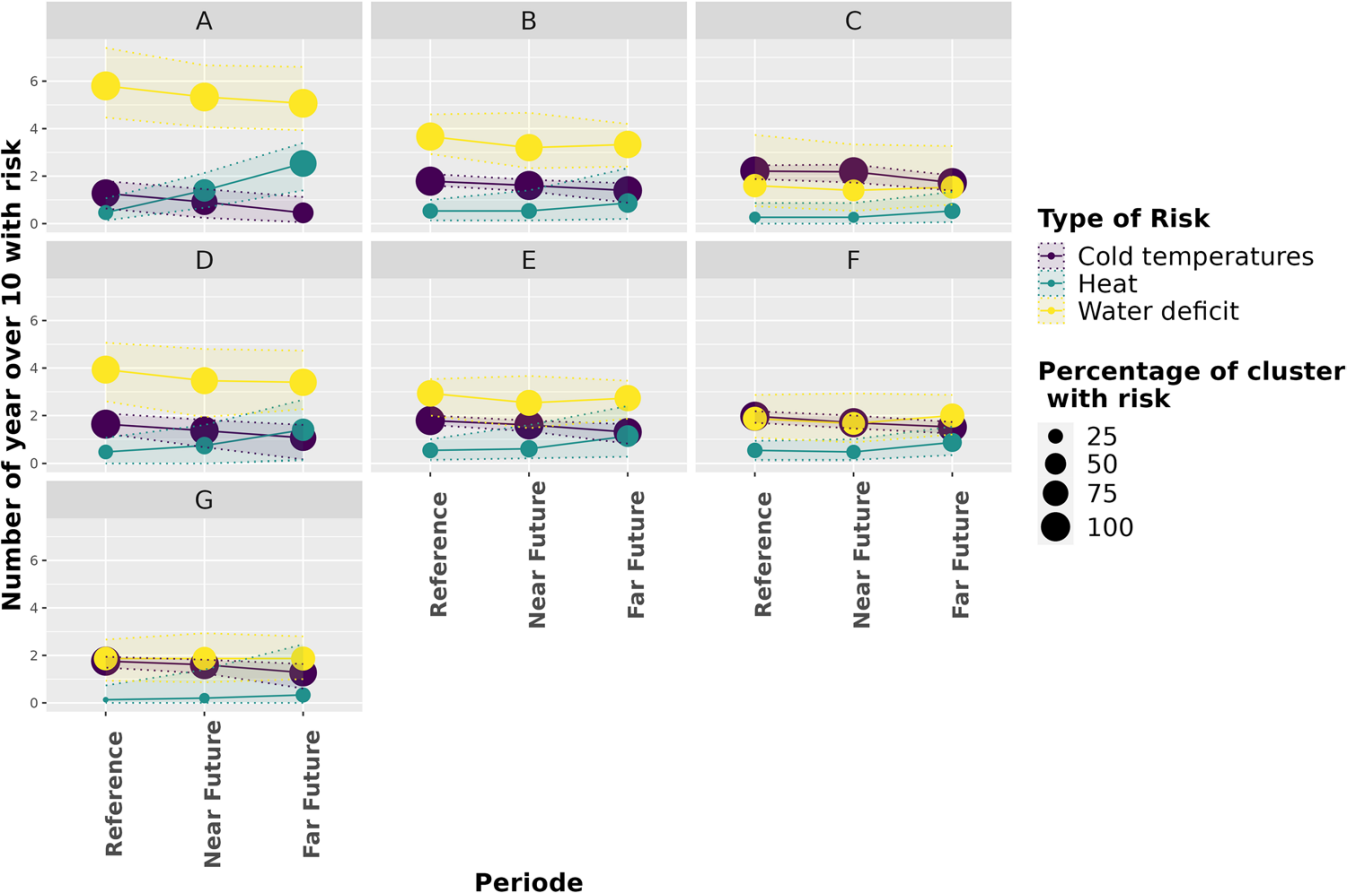
Evolution of the frequency (number of years over a 10-year period) of the different types of climatic risks in the 7 French wheat ecoclimatic zones (WEZs A to G) in the reference (1991–2020), near (2041–2070) and far (2071–2100) future periods under RCP 8.5 scenario. The different types of climatic risks concern cold temperatures, heat and water deficit. The size of the circles corresponds to the percentage of the area of the WEZ affected by the risk. The transparent area delimited by the dotted lines represents the 95 and 5 quantiles of the distribution.

By grouping the different ecoclimatic indicators by type of risk (cold temperatures, heat and water deficit), we highlight changes in their frequency in each WEZ (on the ordinate in Fig. 5 for RCP 8.5, and Supplementary Fig. S5 for RCP 4.5) and their possible spatial expansion or reduction (size of the circles in Figs. 5 and S5). The risk of cold temperature is projected to decrease for both scenarios (especially in the far future), while the frequency and spatial extent of heat stress increase for all wheat ecoclimatic zones and both scenarios, with a threefold increase in the far future for WEZ A under RCP 8.5. The frequency of water deficit, in the WEZs where it is highest in the reference period (A, B, and D), is projected to increase in the near future and then decrease in the far future under RCP 4.5 scenario while it decreases from the near future onwards under RCP 8.5. It remains stable in WEZ G, and decreases in the near future to increase in the far future in WEZ C, E and F under RCP 8.5. Indeed, earlier phenological stages are projected to compensate for the increase in water deficit and allow wheat to avoid periods of drought in the near future under RCP 8.5 and in the far future under RCP 4.5. However, in the far future, with the RCP 8.5 scenario, the phenology of wheat no longer allows it to avoid periods of water deficit in WEZs C, E and F.

Overall, the order of importance (in terms of frequency) of the risks within a WEZ is projected to remain the same in the future, with water deficit being the most frequent in all WEZs, both in the reference period and in the near and far future, except in WEZ C and F where it’s as threatening as cold temperatures. While the main change in the risk of water shortage and cold temperatures is their frequency, heat stress extension also expands, as the percentage of WEZ with this risk increases in the future for all WEZ.

**Figure 6:**
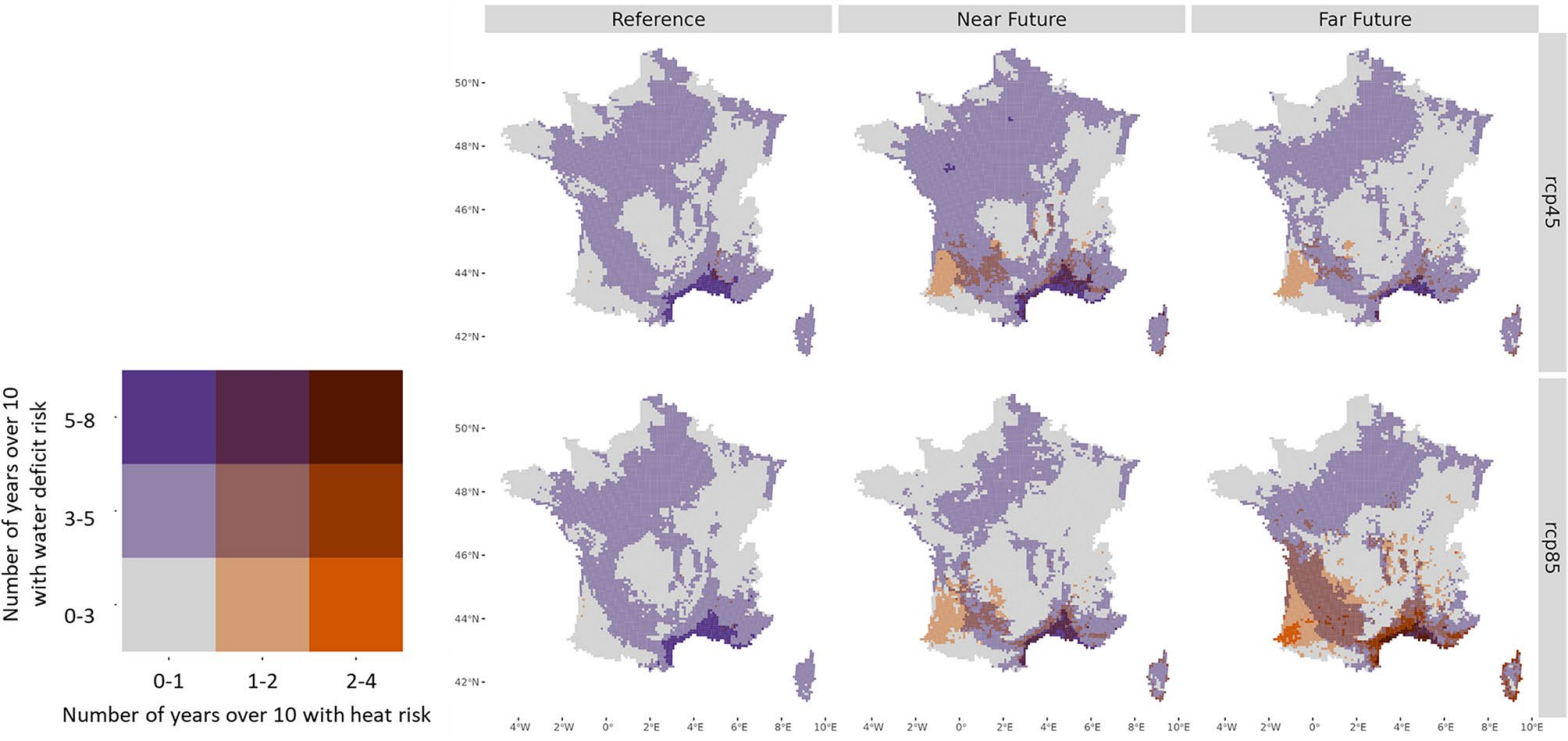
Maps of heat and water deficit risks for different periods, reference (1991–2020), near future (2041–2070) and far future (2071–2100). Each cell of the legend corresponds to a class of heat and/or water deficit risks defined by the number of years with heat and/or water deficit over a 10-year period. The maps correspond to the mean (Q50) risk of the model ensemble for RCP 4.5 (top) and 8.5 (bottom) and (see Supplementary Fig. S7 for the percentiles 5 and 95 maps providing the confidence range of the projected risks).

In order to represent the spatial evolution of the two most important risk factors for wheat in the coming decades in France, heat and water deficit, we combine them in bivariate maps (Fig. 6). Under RCP 4.5, we observe an extension of water deficit in the near future, followed by a withdrawal in the far future driven by the phenological shift, which prevails earlier in the near future under RCP 8.5. The areas affected by heat stress risk will increase in the near future and then in the far future northwards from WEZ A and D for both emission scenarios. The frequency of heat stress also increases, especially in the far future and under RCP 8.5, when it reaches one in three years in extreme southern locations. In the far future, the combination of a high heat risk and a significant water deficit is expected to threaten a larger part of the Mediterranean arc, the Garonne valley and the Corsican coast under RCP 8.5 than with RCP 4.5.

Looking at the evolution by phenological phase (see Supplementary Fig. S6), during the vegetative phase (from emergence to flag leaf), the decrease of water stress risk could be explained by a combination of increased winter precipitation and accelerated phenology. rom flag leaf to grain maturity, a reduced risk of water stress is projected for the near future (Fig. S6b) the Mediterranean region being only affected by water deficit, and an increased risk in the far future (Fig. S6c), particularly in the north of France which was not affected until now (mainly the flag leaf to anthesis phase in the Paris basin and the north-east) as well as in the Garonne valley. Heat stress is projected to become widespread over a large part of the country from the anthesis to the grain maturity stage in the far future, reaching regions that were previously spared.

## Discussion

### General overview

We propose a method to classify agro-climatic risks and their evolution with climate change, combining high-resolution climatic data with a phenological model of wheat. This approach relies on ecoclimatic indicators ^64,65^ and demonstrates the importance of considering phenology to characterise the impact of climate change on crops.

We define a panel of ecoclimatic indicators to characterize wheat vulnerability to climatic factors, and given the spatial variability of both wheat phenology and climatic hazards over the last 30 years (1991–2020), we identify 7 homogeneous wheat ecoclimatic zones (WEZ) according to the climatic risks they are likely to face.

In France, there is a certain degree of spatial stability for the different phenological stages, with initial stages being earlier in the western regions, which then disappears with the progress of the cycle. In the future, the ear at 1 cm stage is expected to be the most affected by warming in mountainous and continental regions, while later stages and in particular the grain maturity stage are expected to advance by two to three weeks in all regions in the far future under the RCP8.5 scenario. These results are consistent with studies in cereals, most of which report a gradual shift of the spring and summer phenophases towards earlier dates over the last 30 years^66^. They also confirm the previously projected evolution of the seasonal timing of the wheat cycle in the future. Gate and Brisson^67^ estimated a mean advance of 5.8 days per degree of annual warming for wheat. This corresponds to wheat crop maturing 16–18 or 21–34 days earlier for a warming of 2.7–3.1 °C in the near future (RCP 4.5–8.5) and 3.7–5.9 °C in the far future (RCP 4.5–8.5), respectively, for France^68^.

Current cold temperatures and water deficit are identified as the most discriminating climatic risks between the WEZs during the early stages, standing out high altitude zones, most continental ones and areas with less pronounced oceanic influence. On the other hand, water deficit and heat stress differentiate the Mediterranean areas from the flag leaf stage onwards.

In the future, heat events are likely to impact wheat crops during later developmental stages despite a shorter crop cycle. By contrast, future water deficit should partially be avoided due to accelerated development in response to warming. It would be interesting to investigate whether this phenomenon could also partly explain why, despite an increase in some weather hazards (especially high temperatures), their impact on yield losses has remained similar in magnitude over recent decades in France^5^, which is not the case in other regions, such as Australia^69^. In particular, our results show that both the extent and frequency of heat stress will increase, while water deficit will decrease only in the near future due to earlier sensitive developmental stages. It is worth noting that in the far future, the combination of water deficit and heat stress, mainly during the reproductive phases of the cycle, will threaten cereal production in the southern half of France, while the northern half will be mostly affected by water deficit, except for the oceanic coast. These results are consistent with previous publications on future climate-related growing conditions for wheat. Trnka et al*.*^70^ showed that, without climate change mitigation (i.e. under RCP 8.5 scenario) nor adaptation, up to 60% of the current wheat-growing area in the world will face simultaneous severe water scarcity events by the end of this century, compared to 15% today. Hristov et al*.*^71^ projected that water deficit will have a negative impact on wheat production in southern Europe, while in the near future (around 2050) the risk of water stress will be lower in the north. By conducting a sensitivity test by shifting the crop phenological date by two weeks, Zhu et al*.*^72^ showed that under RCP4.5 and RCP8.5 in 2070–2099, western Europe will mostly experience heat stress during the vegetative and reproductive periods and low water supply to a lesser extent, while Southern Europe will be vulnerable to low water supply in most of its area during the vegetative period. France is located at the interface between southern Europe, where agricultural production has been projected to be severely reduced by 2050 due to climate change, and northern Europe, where compensation by means of CO_2_ fertilisation could, on the contrary, improve production^9,10,73^. CO_2_ experimental results from a variety of devices (controlled growth chambers, greenhouses, closed-top or open-top chambers, or Free‐Air CO_2_ Enrichment experiments) differ widely from each other and from the results of simulations^74^. Moreover they are scarce for high atmospheric CO_2_ levels, above 700 ppm, whereas the projected atmospheric [CO_2_] level in 2100 is of the order of 1300 ppm. In addition, experiments combining the effects of CO_2_ with high temperatures and water deficits^75^ are still insufficient to clearly identify in which configurations CO_2_ fertilisation and increased water use efficiency compensate for the negative effects of heat and water stress, or when the lower transpiration due to stomatal closure under elevated [CO_2_] hinders the lowering of plant temperature under heat stress. Nevertheless, Helman and Bonfil^76^ recently showed that France (and Germany, two of the three countries with the highest yields per hectare in the world) experienced the worst temperature increases and intense droughts in their wheat-growing areas at the end of the 2010s, enough to wipe out or reverse the gains from rising atmospheric [CO_2_] into significant yield losses.

Our approach takes on added importance in the current context of climate change, which has led to a significant inter-annual and spatial variability in yields. In highly productive countries, such variability accounts for up to 45% of total yield variability^76^. Furthermore, wheat production in Western Europe, and in particular in France, is also expected to become more unstable in the future when considering the evolution of interannual and intraseasonal temperature variability as well as extreme degree-days^77^. Our study predicts a higher frequency of heat and drought risks (sometimes combined in hot spots) in the medium and long term. In line with Liu et al*.*^77^, we argue that the adaptation of wheat production in France should be directed towards solutions aiming at regularity of production in the face of climatic risks, rather than at maximum productivity. This is critical when considering that Europe and the Mediterranean region emerge as particularly vulnerable to wheat crop failures when compared to other breadbasket regions worldwide, increasing the likelihood of synchronous failures globally^78^.

### Perspectives

For the selection of high-risk years, we considered years when the normalised indicator had a value below 0.7, targeting climate effects that alter the physiological response of the plant, regardless of the consequences on final production. Indeed, in this study the choice was made to use an identical threshold (0.7) for all indicators because a single variety was used and the work was carried out on a very large scale, thus assuming equal weight for all risks. That said, especially in the case of early risks, the plant and/or the farmer can adapt (e.g. an early frost that reduces plant density can be compensated by tillering or reseeding). These adaptation strategies are not possible at the end of the cycle, so that late weather hazards are more likely to be associated with yield losses. Taking this type of adaptation into account could be done by adjusting the threshold below which the climatic impact is considered unfavourable, depending on the plant's and/or farmer's ability to adapt. In addition, only one genotype sown at one sowing date is considered in this study, while changing could assist to adapt the crop to get fewer stress in future climates ^12^. Climate models also have limitations, particularly when it comes to projecting climate extremes. Seneviratne et al*.*^79^ point out that projections of changes in temperature extremes tend to be more consistent across climate models (in terms of sign) than for precipitation extremes (wet and dry). The figures presented here are synthetic, reducing both the spatial and temporal dimensions and the variability between climate models. However, it is entirely possible to apply this study to regional approaches^80^, to study an exceptional year on the basis of reference data, or to focus on a critical phase, as the indicators and thresholds are calculated by phase, by cell and for each year, thus demonstrating once again the great genericity of the method. Moreover, large databases on regional agricultural conjunctures at weekly time step are needed to describe crop development and growth, allowing us to assess the robustness of our indicators for large area applications.

In our study, we use a single variety with the average precocity of varieties grown in France. Breeding is one of the major levers for adapting the sector to climate change. However, our study suggests that there are ideotypes of cultivars that would make it possible to avoid certain risks, such as high heat or excessive water deficits, particularly at the end of the cycle. The characteristics of these ideotypes could be targeted according to the specific climatic constraints of the WEZs. The proposed method can thus be used to identify potential locations for wheat breeding trials based on similarity of ecoclimatic indicators (between current ecoclimatic conditions in potential sites and future ecoclimatic conditions in breadbaskets), rather than just climatic conditions ^81,82^. In addition, the construction of water stress indicators that take into account soil characteristics, based on a detailed soil map, would make it possible to better target future impacts and potential adaptations at the local level. Another potential use of these clusters would be to examine whether certain historical clusters move or expand spatially in the future, such as WEZ A moving northwards, as previously demonstrated at the European scale by Ceglar et al*.*^83^ who proceeded a statistical cluster analysis driven by two agro-meteorological indicators based on temperature. Although these authors considered agro-meteorological indicators and crop phenological timing separately, they shed light on the importance of considering dynamic changes in crop exposure to extreme events.

The ecoclimatic indicators we developed incorporate ecophysiological thresholds that reflect a crop tolerance to particular stress. These thresholds may vary between varieties. Our approach could therefore be used to search for resistant or resilient ideotypes, with the value of the tolerance threshold to be reached depending on the time horizon and region considered. To go further, we could include conditional indicators in the method that would take into account the non-additive effects of successive repeated stressful events (e.g. in Ref.^84^). The combination of indicators, as previously done for maize^64^, would allow consideration of the multiple effects of stresses and their interactions within a single phenological phase, or even provide a synthetic index of climate suitability for a given area.

A complementary socio-economic approach would make it possible to better characterise and justify the decisive threshold to determine whether risk is critical (in our study, normalised indicator below 0.7). For example, these thresholds might be set according to criteria such as the expected economic return or the farmer's risk aversion. Our approach could then be integrated into the process of co-constructing strategies with the sector to adapt wheat breadbaskets to climate change.

The designed methodology presented in this article is relevant not only for wheat producers in France but also for agricultural systems worldwide. It provides a significant advance in our understanding of the spatial dynamics of climate risk in agriculture, and has the potential to inform more effective adaptation strategies to maintain crop production and resilience in the face of climate change.

### Supplementary Information

Supplementary Information.

## Data Availability

All the data used to produce the analysis and figures are available using this private link : https://entrepot.recherche.data.gouv.fr/privateurl.xhtml?token=860643c7-758f-4f32-a6fc-01a82e9fe5b6. All the code to produce the analysis and figure are available here : https://forgemia.inra.fr/renan.le-roux/wheatclimaterisk. All analyses and figures were performed using R Statistical Software^85^, the tidyverse^86^ and ggplot2^87^ packages.
