# Supplementary material for: Mapping the race between crop phenology and climate risks for wheat in France under climate change
Source: Sci Rep. 2024 Apr 8;14:8184. doi: 10.1038/s41598-024-58826-w (PMC11001926; doi:10.1038/s41598-024-58826-w)
Supplement: Supplementary file 1 — Supplementary Information. [file 41598_2024_58826_MOESM1_ESM.docx]

**Mapping the race between crop phenology and climate risks for wheat in France under climate change**

**Author list:**

Renan Le Roux^^[[1]](#footnote-2)^^*, Alexis Durand^1^, Jean-Charles Deswarte^^[[2]](#footnote-3)^^, Marie-Odile Bancal^^[[3]](#footnote-4)^^, Karine Chenu^^[[4]](#footnote-5)^^, Nathalie de Noblet-Ducoudré^^[[5]](#footnote-6)^^, Burak Bulut^5^, Carina Furusho-Percot^1^, Iñaki García de Cortázar-Atauri^1^, Olivier Maury^1^, Jérémie Décome^1^, Marie Launay^1^*.

* Corresponding authors: Le Roux, R. ([renan.le-roux@inrae.fr](mailto:renan.le-roux@inrae.fr),ORCID iD 0000-0001-7778-1878)  & Launay M. ([marie.launay@inrae.fr](mailto:marie.launay@inrae.fr); ORCID iD 0000-0003-4067-3907)

**Figure S1.** Normalization functions: (a) Modified exponential (shown here with the specific coefficients applied for the indicator of the number of days with very cold temperatures leading to mortality between emergence and ear 1 cm stages), (b) Inverse sigmoid (shown here with the specific coefficients applied for the indicator of heat days damaging grain filling between anthesis and grain maturity), (c) Sigmoid (shown here with the specific coefficients applied for the indicator of vernalizing days between emergence and ear 1 cm stages)


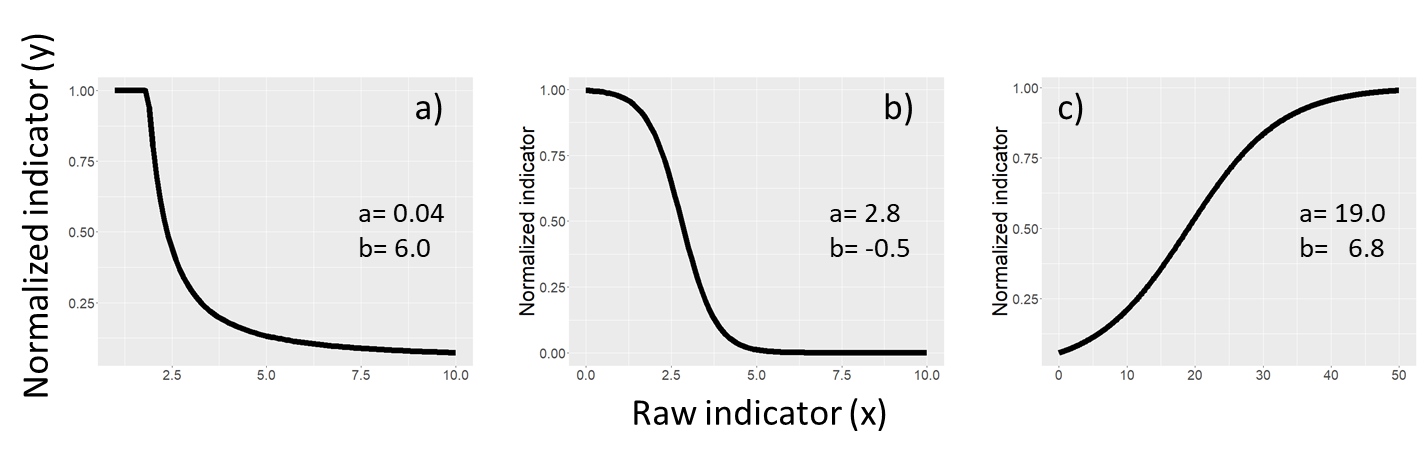


Sigmoid function:

$$y=\frac{1}{1+e^{\frac{\left( a-x \right)}{b}}}$$

Modified exponential function:

$$y=min\left( a\times e^{\frac{b}{x}},1 \right)$$

**Table S1.** List of key climatic effects considered during successive phenological sensitive periods of the wheat development, and the corresponding ecoclimatic indicators with their associated normalization function. PET refers to the reference evapotranspiration (mm) calculated from a well-watered, full-cover grass surface with the Penman–Monteith formula in Ref.[82](https://paperpile.com/c/3vaRbJ/7penc). To be consistent with the phenological model assessing the vernalisation effect on development, the minimum temperature for a day to be vernalising was set at 0°C, and the maximum one at 13°C.

| **Phenological sensitive period** | **Climatic effect** | **Ecoclimatic indicator** | | | **Normalisation function** | | **Comment on the normalisation effect** | **Ref.** |
| --- | --- | --- | --- | --- | --- | --- | --- | --- |
| *starting to ending stages  (BBCH) name* |  | *definition* | *base weather variable and threshold* | *Unit* | *function* | *coefficients* |  |  |
| 15 days before Sowing to Emergence (ante 00 → 09) ante SO-EM | water deficit | Cumulative rainfall | daily rainfall | mm | sigmoid | a=22.0; b=10.0 | null effect since 60 mm cumulated rainfall | ^32^ |
|  |  |  |  |  |  |  |  |  |
| Sowing to Emergence (00 → 09) SO-EM | waterlogging | Number of rainy days in excess | daily rainfall > 30 mm | days | exponential | a=0.026; b=6.63 | medium effect for a 2 days flooding event, major effect for 3 days and lethality for 5 days | ^30^ |
|  | cold temperature | Number of cold days | daily average temperature < 2°C | days | sigmoid | a=9; b=-2 | stronger effect from 5 to 12 days | ^33, 36^ |
|  | frost | Number of frost days | daily minimum temperature < -5°C | days | exponential | a=0.1; b=3.5 | nearly null effect for 1 day, lethal effect for 5 days | ^34^ |
|  |  |  |  |  |  |  |  |  |
| Emergence to Ear 1 cm (10 → 30) EM-E1 | water deficit | Cumulative sum of daily (rainfall - PET) | daily rainfall, daily PET | mm | sigmoid | a=290; b=40 | rapid decline below the critical 290 mm threshold | ^55^ |
|  | cold temperature | Number of cold days | daily average temperature < 5°C | days | exponential | a=0.51; b=1.31 | medium effect since 10 cold days | ^35^ |
|  | frost | Number of frost days | daily minimum temperature < -8°C | days | exponential | a=0.04; b=6=6 | nearly null values for 4 to 6 very cold days | ^36^ |
|  | vernalization | Number of vernalizing days | daily average temperature (minimal threshold 0°C; maximal threshold 13°C) | days | sigmoid | a=19.0; b=6.8 | no more effect since the indicator is equal to the cultivar cold requirements | ^28, 37-40^ |
|  | devernalization | Number of devernalizing days | daily average temperature > 20°C | days | sigmoid | a=8.0; b=-2.0 | decline from 2 days and null value since 15 days | ^41^ |
|  |  |  |  |  |  |  |  |  |
| **Phenological sensitive period** | **Climatic effect** | **Ecoclimatic indicator** | | | **Normalisation function** | | **Comment on the normalisation effect** | **Ref.** |
| *starting to ending stages  (BBCH) name* |  | *definition* | *base weather variable and threshold* | *Unit* |  |  |  |  |
| Ear 1 cm to Flag leaf (31 → 39) E1-FL | heat | Number of warm nights | daily minimum temperature > 12°C | days | sigmoid | a=20; b=-4 | low effect of 10 warm nights, medium of 20, strong with 40 | ^34, 42-44^ |
|  | cold temperature | Number of cold days | daily minimum temperature < 4°C | days | exponential | a=0.55; b=1.23 | medium value (0.6) since 10 cold days | ^21, 34^ |
|  | frost | Number of frost days | daily minimum temperature < -5°C | days | sigmoid | a=2.8; b=-0.5 | rapid decline after 2 days, null value since 5 days | ^36^ |
|  | water deficit | Cumulative sum of daily (rainfall - PET) | daily rainfall, daily PET | mm | sigmoid | a=-130; b=40 | rapid decline below 130 mm of cumulated deficit | ^55^ |
|  |  |  |  |  |  |  |  |  |
| \| 10 days before Flag leaf  to 10 days after (≈ 39) 20aroundFL \| \| --- \| | low radiation | Number of days with low radiation | daily global radiation < 200W/m² | days | sigmoid | a=10.0; b=-2.0 | null value since 20 days | ^21^ |
|  | cold temperature | Number of cold days | daily minimum temperature < 4°C | days | sigmoid | a=10.0; b=-2.0 | null value since 20 days (full period) | ^21, 30^ |
|  |  |  |  |  |  |  |  |  |
| Flag leaf to Anthesis(40 → 64)FL-AN | cold temperature | Number of cold days | daily minimum temperature < 4°C | days | sigmoid | a=10.0; b=-2.0 | null value since 20 days | ^21, 34^ |
|  | very cold temperature | Number of very cold days | daily average temperature < 0°C | days | exponential | a=0.09; b=2.1 | rapid decline from 1 day, null value since 5 days | ^45, 46^ |
|  | heat | Number of warm days | daily maximum temperature > 30°C | days | sigmoid | a=3.0; b=-0.5 | null value since 5 days | ^34, 47-50^ |
|  | water deficit | Cumulative sum of daily (rainfall - PET) | daily rainfall, daily PET | mm | sigmoid | a=-140; b=40 | rapid decline below 140 mm of cumulated deficit | ^56, 57^ |
|  |  |  |  |  |  |  |  |  |
| \| Anthesis to Grain maturity (65 → 89) AN-GM \| \| --- \| \| | very cold temperature | Number of very cold days | daily minimum temperature < -2°C | days | sigmoid | a=2.8; b=-0.5 | rapid decline from 2 days, null value since 5 days | ^54^ |
|  | heat | Number of warm days | daily maximum temperature > 30°C | days | sigmoid | a=2.8; b=-0.5 | rapid decline from 2 days, null value since 5 days | ^47, 51-53^ |
|  | water deficit | Cumulative sum of daily (rainfall - PET) | daily rainfall, daily PET | mm | sigmoid | a=-320; b=40 | rapid decline below 320 mm of cumulated deficit | ^55, 56, 58-61^ |

**Figure S2.** Example of indicator aggregation by major risk family to obtain a 30-year value


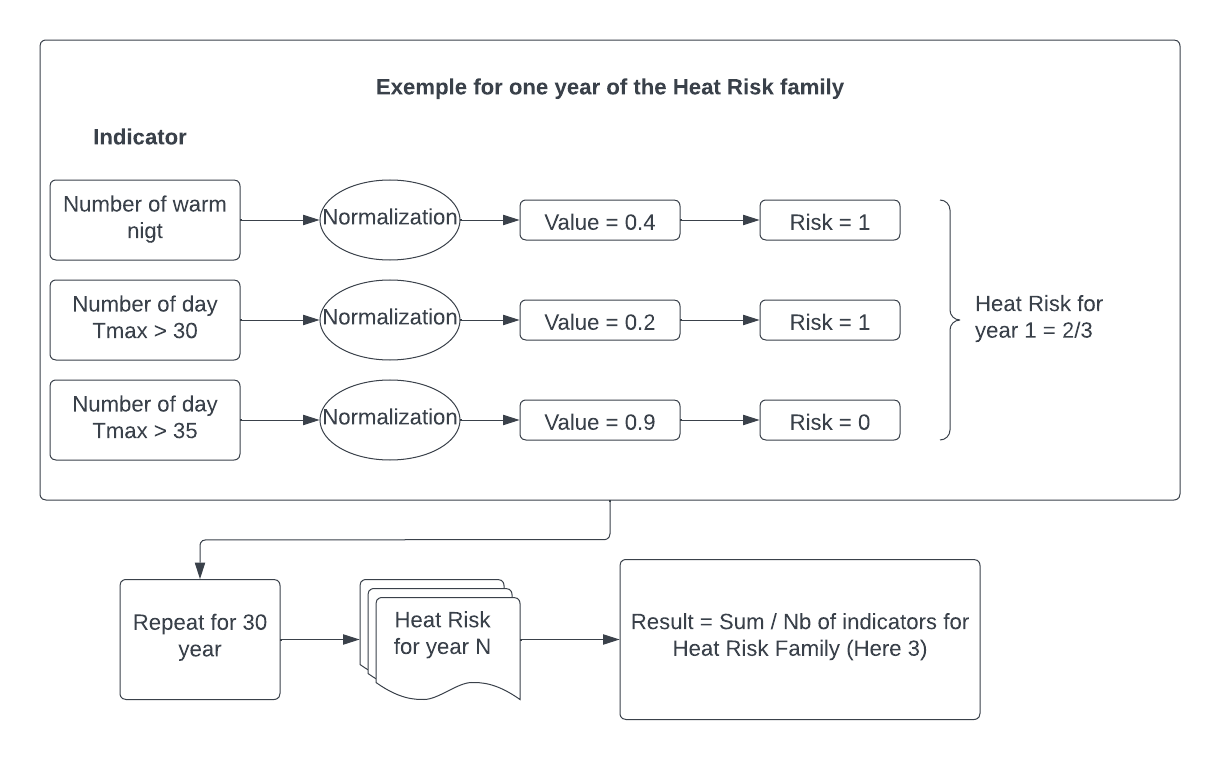


**Figure S3.** Simulated vs. observed dates of phenological stages ear 1 cm (green dots), flag leaf (orange dots) and anthesis (purple dots). Each point represents a site x year observation from the EPIPHYT database (<https://data.pheno.fr/>). Black continue line represents the 1:1 relationship.


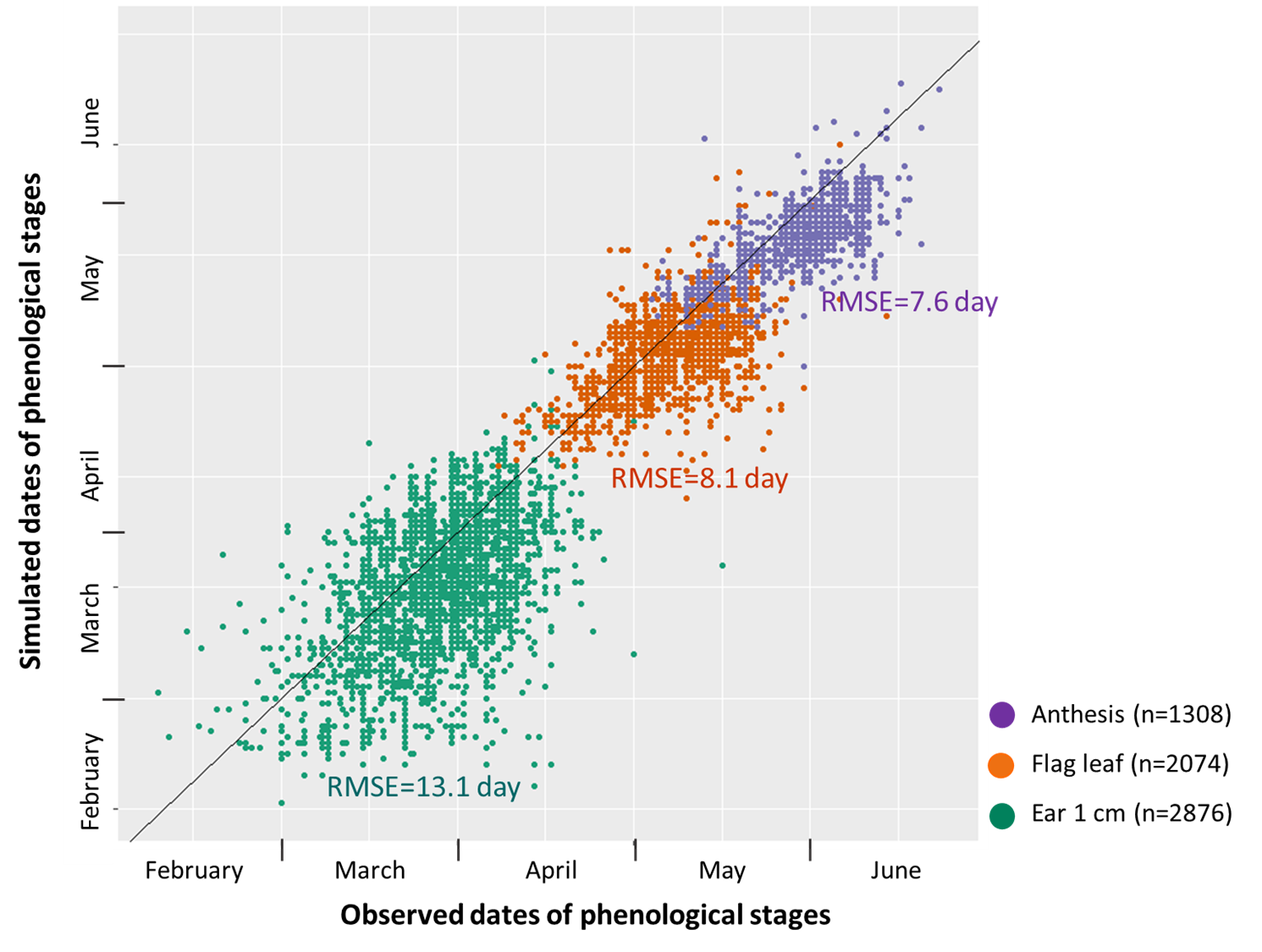


**Equations of the phenological model.**

Development time is calculated by accumulating the daily thermal time increment ${dev}_{unit}$ according to the growing degree days approach (GDD in °Cdays), slowed by reduction factors according to photoperiod conditions, and vernalisation requirements:

$dev_{unit}\left( d \right)=\left( T_{air}-T_{base} \right)\cdot rfp\left( d \right)\cdot rfv\left( d \right)$ (1)

where ${dev}_{unit}$ is the daily thermal time, $T_{air}$ is the daily average air temperature, $T_{base}$ is the base temperature of 0◦C for wheat, $rfp$ and $rfv$ are the daily reduction factors for photoperiod and vernalisation effects (respectively equations (2) and (3)).

$rfp\left( d \right)=\frac{\left( phoi\left( d \right)-phobase \right)}{phosat-phobase}$ (2)

where $phoi$ is the daily current photoperiod, $phobase$ and $phosat$ are respectively the lower and higher photoperiod thresholds (6.3 and 20.0 for wheat).

$rfv\left( d \right)=\frac{\sum_{i=EM}^{d} jvi\left( i \right)-nvmini}{nvcult-nvmini}$ and $jvi\left( d \right)=max\left( 1-\left( \frac{tcold-T_{air}\left( d \right)}{amp_{cold}} \right)^{2};0.0 \right)$ (3)

where $jvi$ is the vernalising value of a given day, $nvmini$ is the minimum number of vernalising days that are required (equal to 7 days), $nvcult$ is the genotype-dependent number of vernalising days (equal to 55 days for Talent wheat variety), $tcold$ is the optimum vernalisation temperature (6.5°C), and ${amp}_{cold}$ is a parameter indicating the species sensitivity to vernalization and corresponding to the thermal half-amplitude around the optimal temperature for vernalization (10°C). The $rfp$ and $rfv$ reduction factors are no longer effective after flowering. For more details, please refer to:

<https://w3.avignon.inra.fr/forge/attachments/download/3127/STICS-SOIL-CROP-MODEL_V10.0.html>

**Figure S4.** Reference period (1991-2020), near (2041-2070) and far (2071-2100) futures’ distribution of wheat main phenological phases (EM-E1: emergence to ear 1 cm; E1-FL: ear 1 cm to flag leaf; FL-AN: flag leaf to anthesis; AN-GM: anthesis to grain maturity) under RCP 4.5 scenario in the 7 French wheat ecoclimatic zones (WEZs A to G). DOY: day of the year.


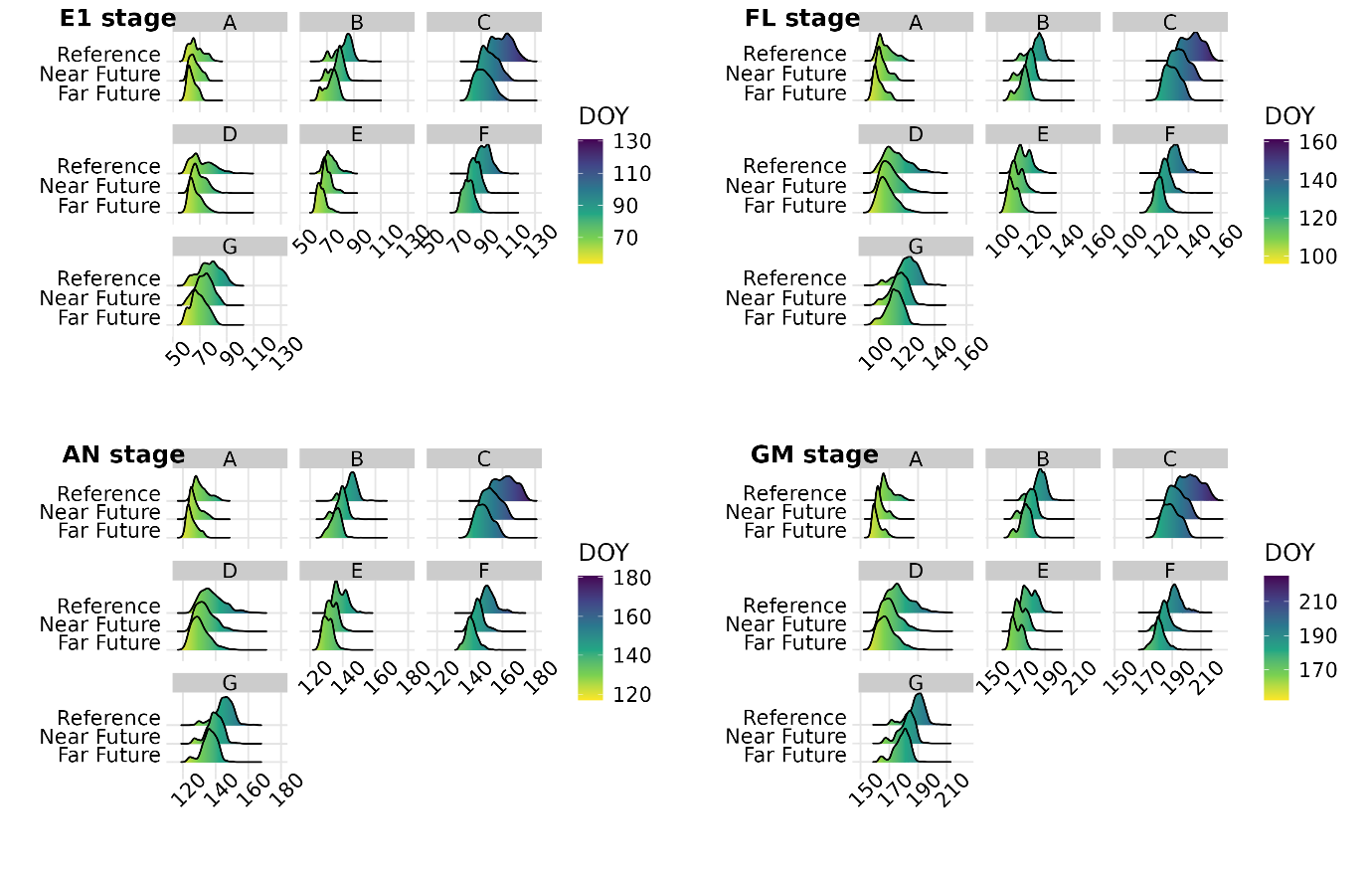


**Table S2.** Median of the simulated dates of phenological stages per Wheat Ecoclimatic Zone in France, for RCP 4.5 and 8.5 scenarios, and reference/historical period (1991-2020), near future (2041-2070) and far future (2071-2100) periods.

| NamePhase | Name | Reference | Near Future | Far Future | diff_near_future | diff_far_future | scenario |
| --- | --- | --- | --- | --- | --- | --- | --- |
| AN stage | A | 129 | 123 | 119 | -6 | -10 | rcp85 |
| AN stage | B | 145 | 136 | 129 | -8.5 | -15.5 | rcp85 |
| AN stage | C | 162.5 | 149 | 139 | -13 | -23 | rcp85 |
| AN stage | D | 138.5 | 131 | 125 | -8 | -14 | rcp85 |
| AN stage | E | 141 | 133 | 127 | -8 | -14 | rcp85 |
| AN stage | F | 151 | 141 | 133 | -10 | -18 | rcp85 |
| AN stage | G | 145 | 137 | 130 | -8 | -15 | rcp85 |
| E1 stage | A | 63 | 64 | 61 | 0 | -3 | rcp85 |
| E1 stage | B | 84 | 74 | 66 | -10 | -17 | rcp85 |
| E1 stage | C | 107 | 90 | 78 | -16.5 | -29.5 | rcp85 |
| E1 stage | D | 75 | 68 | 63 | -6 | -12.5 | rcp85 |
| E1 stage | E | 74 | 67 | 62 | -7 | -13 | rcp85 |
| E1 stage | F | 93.5 | 81 | 71 | -12 | -22 | rcp85 |
| E1 stage | G | 76 | 68 | 63 | -8 | -13.5 | rcp85 |
| FL stage | A | 107.5 | 103 | 100 | -4.5 | -8 | rcp85 |
| FL stage | B | 125 | 116 | 110 | -9 | -15 | rcp85 |
| FL stage | C | 143 | 129 | 120 | -13 | -22.5 | rcp85 |
| FL stage | D | 118 | 110 | 105 | -7.25 | -13.25 | rcp85 |
| FL stage | E | 119 | 111 | 106 | -8 | -13.5 | rcp85 |
| FL stage | F | 131.5 | 122 | 114 | -10 | -17.5 | rcp85 |
| FL stage | G | 123 | 114 | 109 | -8 | -14 | rcp85 |
| GM stage | A | 167 | 160 | 154 | -7 | -13 | rcp85 |
| GM stage | B | 186 | 176 | 168 | -10 | -18 | rcp85 |
| GM stage | C | 204 | 189 | 179 | -14 | -24.5 | rcp85 |
| GM stage | D | 177.75 | 168.5 | 161 | -9 | -17 | rcp85 |
| GM stage | E | 182.5 | 173 | 165 | -10 | -18 | rcp85 |
| GM stage | F | 192 | 181 | 172 | -11 | -20 | rcp85 |
| GM stage | G | 190 | 180 | 172 | -9.5 | -18 | rcp85 |
| AN stage | A | 130 | 127 | 125 | -3 | -5 | rcp45 |
| AN stage | B | 145 | 139 | 136 | -5.5 | -9 | rcp45 |
| AN stage | C | 162 | 153 | 148 | -9 | -14 | rcp45 |
| AN stage | D | 136.75 | 133 | 130 | -4 | -7 | rcp45 |
| AN stage | E | 138 | 133 | 131 | -5 | -8 | rcp45 |
| AN stage | F | 151 | 145 | 141 | -6 | -10 | rcp45 |
| AN stage | G | 145.5 | 140 | 137 | -6 | -9 | rcp45 |
| E1 stage | A | 66 | 65 | 63 | -1 | -3 | rcp45 |
| E1 stage | B | 84 | 79 | 74 | -5 | -9 | rcp45 |
| E1 stage | C | 106.5 | 96 | 91 | -10 | -14.5 | rcp45 |
| E1 stage | D | 69 | 68 | 65 | -1 | -4.5 | rcp45 |
| E1 stage | E | 72 | 69 | 65 | -2.5 | -7 | rcp45 |
| E1 stage | F | 93 | 85 | 81 | -7 | -12 | rcp45 |
| E1 stage | G | 77 | 73 | 69 | -4 | -8 | rcp45 |
| FL stage | A | 108 | 106 | 104 | -2 | -4 | rcp45 |
| FL stage | B | 125 | 120 | 117 | -5 | -8 | rcp45 |
| FL stage | C | 143 | 134 | 130 | -8 | -13 | rcp45 |
| FL stage | D | 115 | 112 | 109 | -3 | -5.5 | rcp45 |
| FL stage | E | 116 | 112 | 109 | -4 | -7 | rcp45 |
| FL stage | F | 131.5 | 125 | 122 | -6 | -10 | rcp45 |
| FL stage | G | 123.5 | 118 | 115 | -5 | -8 | rcp45 |
| GM stage | A | 168 | 163 | 161 | -5 | -7 | rcp45 |
| GM stage | B | 186.5 | 180 | 176 | -7 | -10 | rcp45 |
| GM stage | C | 203 | 193 | 189 | -10 | -14 | rcp45 |
| GM stage | D | 176 | 170 | 167 | -6 | -9 | rcp45 |
| GM stage | E | 179 | 173 | 170 | -6 | -9 | rcp45 |
| GM stage | F | 192 | 184 | 181 | -7 | -11 | rcp45 |
| GM stage | G | 189 | 183 | 180 | -6.5 | -10 | rcp45 |

**Figure S5.** Evolution of the frequency (number of years over a 30-year period) of the different types of climatic risks since reference period (1991-2020), near (2041-2070) and far (2071-2100) futures in the 7 French wheat ecoclimatic zones (WEZs A to G) under RCP 4.5 scenario. The different types of climatic risks concern cold temperatures, excess water, heat and drought. The size of the circles corresponds to the percentage of the area of the WEZ affected by the risk. The transparent area delimited by the dotted lines represents the 95 and 5 quantiles of the distribution.


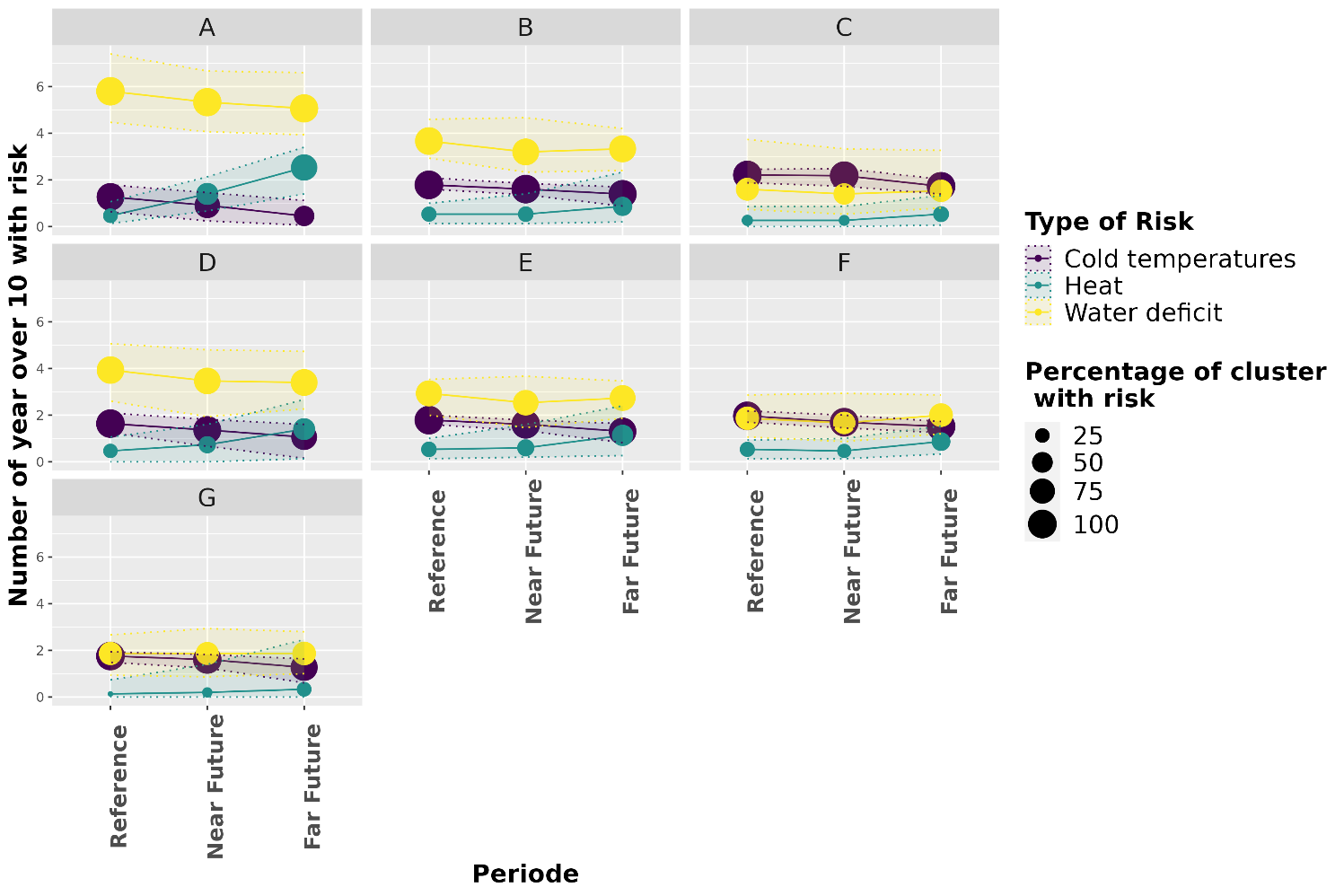


**Figures S6.** Bivariate maps of heat and water deficit indicators per phenological phase

Figure S6.1. Bivariate maps of heat and water deficit indicators per phenophase for different periods for RCP 8.5. A) represents historical (1991-2020), B) near future (2041-2071) and C) far future (2071-2100). EM-E1: emergence to ear 1cm stage; E1-FL: ear 1cm to flag leaf stage; FL-AN: flag leaf to anthesis stage; AN-GM: anthesis to grain maturity stage. Each cell of the legend corresponds to a class of heat and water deficit combined risk and corresponds to the median of the three models.


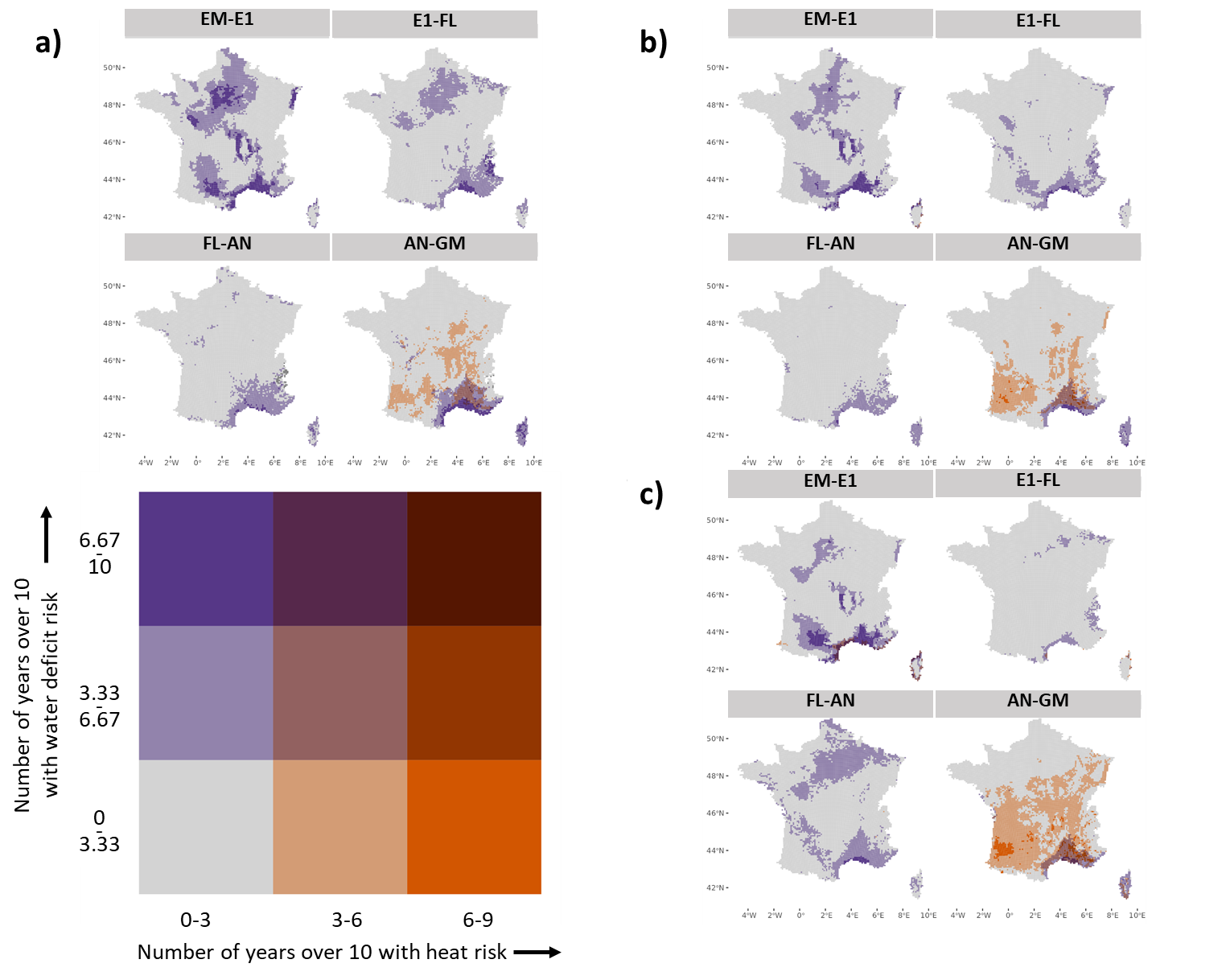


Figure S6.2. Bivariate maps of heat and water deficit indicators per phenophase for different periods for RCP 4.5. A) represents historical (1991-2020), B) near future (2041-2071) and C) far future (2071-2100). EM-E1: emergence to ear 1cm stage; E1-FL: ear 1cm to flag leaf stage; FL-AN: flag leaf to anthesis stage; AN-GM: anthesis to grain maturity stage. Each cell of the legend corresponds to a class of heat and water deficit combined risk and corresponds to the median of the three models.


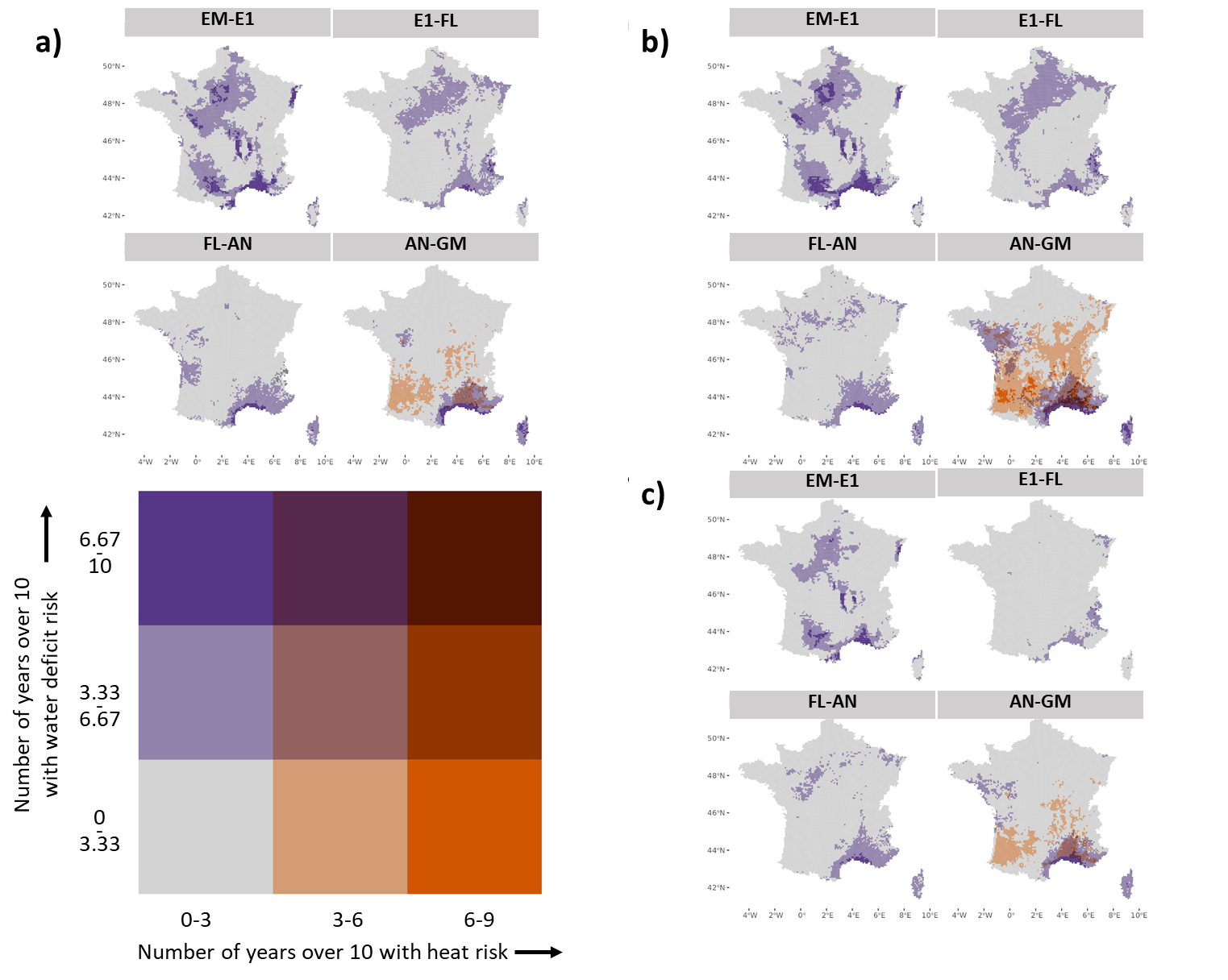


**Figure S7.** Bivariate Q5 and Q95 maps of heat and water deficit indicators for different periods for RCP 4.5. and RCP 8.5 maps, associated with quantiles 0.05 and 95 of the three models, for reference period (1991-2020), future (2041-2071) and far future (2071-2100). Each cell of the legend corresponds to a class of heat and water deficit combined risk.


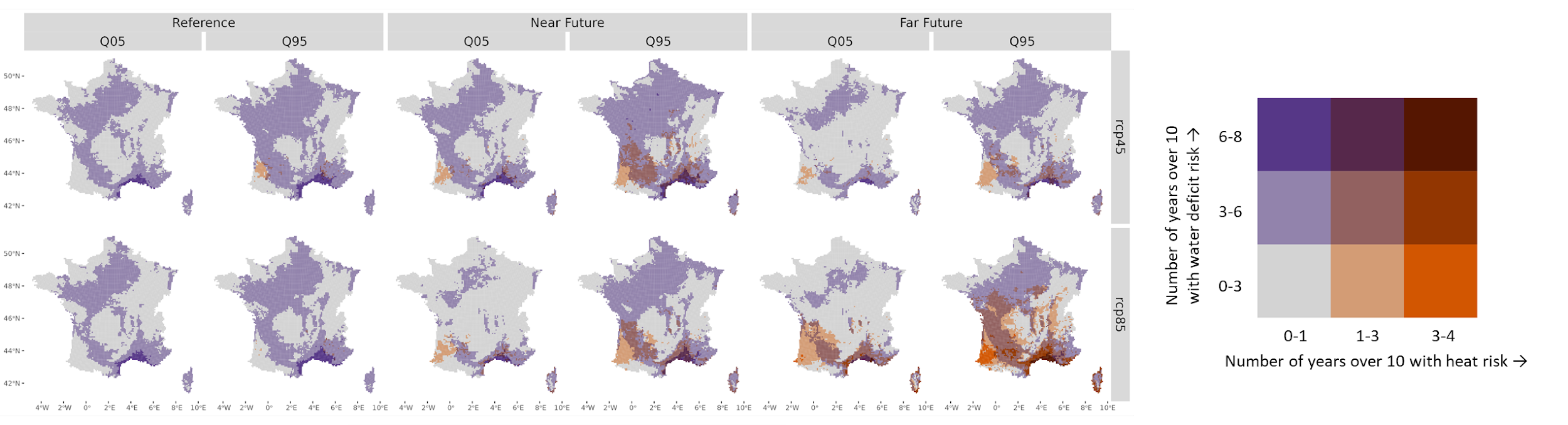

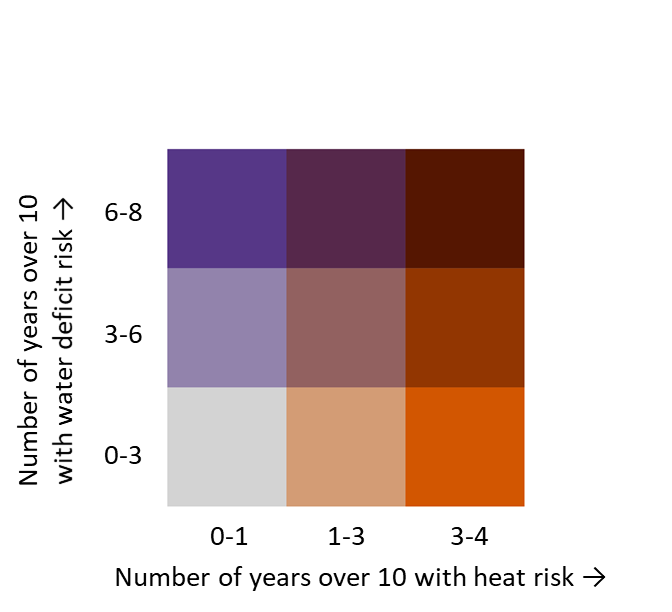


1. INRAE, US AgroClim, F-84914, Avignon, France [↑](#footnote-ref-2)
2. ARVALIS - Institut du Végétal, Villiers-le- Bâcle, France [↑](#footnote-ref-3)
3. Université Paris-Saclay, INRAE, UMR Ecosys Agroparistech, F-78850, Thiverval-Grignon, France [↑](#footnote-ref-4)
4. The University of Queensland, Queensland Alliance for Agriculture and Food Innovation (QAAFI), Leslie Research Facility, 13 Holberton street, Toowoomba, QLD 4350, Australia [↑](#footnote-ref-5)
5. Laboratoire des Sciences du Climat et de l`Environnement LSCE/IPSL, Unité Mixte CEA-CNRS-UVSQ, Université Paris-Saclay, 91191 Gif-Sur-Yvette, France [↑](#footnote-ref-6)
